# Opening the door to greater phylogeographic inference in Southeast Asia: Comparative genomic study of five codistributed rainforest bird species using target capture and historical DNA

**DOI:** 10.1002/ece3.5964

**Published:** 2020-03-06

**Authors:** Haw Chuan Lim, Subir B. Shakya, Michael G. Harvey, Robert G. Moyle, Robert C. Fleischer, Michael J. Braun, Frederick H. Sheldon

**Affiliations:** ^1^ Department of Biology George Mason University Fairfax Virginia; ^2^ Department of Vertebrate Zoology National Museum of Natural History Smithsonian Institution Washington District of Columbia; ^3^ Center for Conservation Genomics Smithsonian Conservation Biology Institute Washington District of Columbia; ^4^ Museum of Natural Science and Department of Biological Sciences Louisiana State University Baton Rouge Louisiana; ^5^ Department of Ecology and Evolutionary Biology University of Tennessee Knoxville Tennessee; ^6^ Biodiversity Institute and Department of Ecology and Evolutionary Biology University of Kansas Lawrence Kansas

**Keywords:** Indochina, Isthmus of Kra, population genetics, rainforest birds, Sundaland, ultraconserved elements

## Abstract

Indochina and Sundaland are biologically diverse, interconnected regions of Southeast Asia with complex geographic histories. Few studies have examined phylogeography of bird species that span the two regions because of inadequate population sampling. To determine how geographic barriers/events and disparate dispersal potential have influenced the population structure, gene flow, and demographics of species that occupy the entire area, we studied five largely codistributed rainforest bird species: *Arachnothera longirostra*, *Irena puella*, *Brachypodius atriceps*, *Niltava grandis*, and *Stachyris nigriceps*. We accomplished relatively thorough sampling and data collection by sequencing ultraconserved elements (UCEs) using DNA extracted from modern and older (historical) specimens. We obtained a genome‐wide set of 753–4,501 variable loci and 3,919–18,472 single nucleotide polymorphisms. The formation of major within‐species lineages occurred within a similar span of time (0.5–1.5 mya). Major patterns in population genetic structure are largely consistent with the dispersal potential and habitat requirements of the study species. A population break across the Isthmus of Kra was shared only by the two hill/submontane insectivores (*N. grandis* and *S. nigriceps*). Across Sundaland, there is little structure in *B. atriceps*, which is a eurytopic and partially frugivorous species that often utilizes forest edges. Two other eurytopic species, *A. longirostra* and *I. puella*, possess highly divergent populations in peripheral Sunda Islands (Java and/or Palawan) and India. These species probably possess intermediate dispersal abilities that allowed them to colonize new areas, and then remained largely isolated subsequently. We also observed an east–west break in Indochina that was shared by *B. atriceps* and *S. nigriceps*, species with very different habitat requirements and dispersal potential. By analyzing high‐throughput DNA data, our study provides an unprecedented comparative perspective on the process of avian population divergence across Southeast Asia, a process that is determined by geography, species characteristics, and the stochastic nature of dispersal and vicariance events.

## Graphical abstract

Phylogeography of taxa occupying Southeast Asia is poorly known despite its importance as a storehouse of tropical biological diversity. We use a combination of sequence capture, high‐throughput sequencing, and historical samples to conduct a region‐wide, multispecies comparative study that unveils phylogeographic patterns with unprecedented genetic resolution.

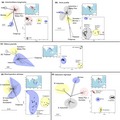

## 1 INTRODUCTION

Avian biogeography in continental Southeast Asia, an area including the mainland and continental islands, has a long history of study (Deignan, 1945; Hughes, Round, & Woodruff, 2003; Smythies, 1953, 1960; Wells, 2007). This base of knowledge has been augmented in the last 15 years by a steady stream of molecular phylogenetic reconstructions that have identified a complex pattern of colonization into, out of, and within the region (e.g., Moyle, Andersen, Oliveros, Steinheimer, & Reddy, 2012; Oliveros, Field, et al., 2019; Wang, Kimball, Braun, Liang, & Zhang, 2017), and which have substantially improved Southeast Asian bird classification (Cai et al., 2019; Cibois et al., 2018; Cibois, Kalyakin, Han, & Pasquet, 2002; Fuchs, Pasquet, Couloux, Fjeldså, & Bowie, 2009; Lim et al., 2019; Moyle et al., 2012; Sangster, Alström, Forsmark, & Olsson, 2010; Shakya & Sheldon, 2017; Zhang et al., 2016). However, phylogenetic studies are imprecise when it comes to identifying the drivers of diversification and extinction, such as changes in gene flow and population sizes, because they span large temporal and spatial scales. On the other hand, phylogeographic investigations within species and species groups provide a better understanding of proximate mechanisms of avian diversification, spatial structuring of genetic diversity, and even adaptive variation within species (Rissler, 2016). Focusing genetic sampling within species and on recent evolutionary history also produces better data for resolving the effects of environmental, geographic, and geological changes on populations. Unlike phylogenetic studies, however, phylogeographic studies of Southeast Asian birds are still relatively rare and usually limited in geographic scope, largely because of inadequate availability of population samples for comparison. The general lack of geographically comprehensive datasets from multiple taxonomic groups (e.g., plants, insects, mammals) has hindered our ability to compare and synthesize information into a complete picture of conditions and factors that have shaped population structure in this important tropical region.

In Indochina, avian population research has been concentrated mainly on the eastern extension of the Himalayans and the southwestern Chinese mountain systems (Fuchs, Ericson, & Pasquet, 2008; Liu et al., 2012; Päckert et al., 2012; Qu et al., 2015; Zou, Lim, Marks, Moyle, & Sheldon, 2007), and on geographic variation and taxonomy at local levels (Fuchs & Zuccon, 2018; Garg et al., 2016; Mahood et al., 2013). Only a few studies have investigated species structure across the entirety of Indochina (e.g., Dong et al., 2014; Fuchs, Ericson, Bonillo, Couloux, & Pasquet, 2015; Round et al., 2017) or across the Isthmus of Kra, which joins Indochina and Sundaland (Dejtaradol et al., 2016; Lim et al., 2019; Manawatthana, Laosinchai, Onparn, Brockelman, & Round, 2017), and all of these studies have suffered from insufficient geographic sampling. In Sundaland, phylogeographic research on birds has focused primarily on Borneo, where sampling is relatively good compared to the Malay Peninsula, Sumatra, and Java (Sheldon, Lim, & Moyle, 2015). Sundaic research has emphasized the genetic break between eastern and western populations of lowland species (Lim et al., 2017, 2010; Lim, Rahman, Lim, Moyle, & Sheldon, 2011) and relationships of populations in Bornean mountains (Chua et al., 2017; Gawin et al., 2014; Manthey et al., 2017; Moyle, Schilthuizen, Rahman, & Sheldon, 2005).

Almost all avian phylogeographic research in Southeast Asia has relied on mitochondrial DNA (mtDNA) comparisons (except Garg et al., 2016; Gwee et al., 2019; Lim et al., 2017; Manthey et al., 2017). Some studies have also compared a small number of Sanger‐sequenced nuclear genes, but these rarely provide much information at the population level. Although mtDNA has many strengths, such as simple maternal inheritance and a rapid rate of evolution (Tamashiro et al., 2019; Zink & Barrowclough, 2008), it can yield inaccurate phylogeographic inferences, as has recently been demonstrated by three genomic studies of Bornean bird populations (Campillo, Oliveros, Sheldon, & Moyle, 2018; Lim et al., 2017; Manthey et al., 2017) that revisited earlier mtDNA studies (Gawin et al., 2014; Lim et al., 2011; Moyle et al., 2011). More importantly, as a single locus, mtDNA cannot provide much insight into key population genetic parameters, such as gene flow, genetic admixture within individuals, and timing of population (vs. gene) divergence (Ballard & Whitlock, 2004). The increased application of strategies for obtaining highly multilocus genomic datasets promises to yield more accurate and detailed phylogeographic inference from Southeast Asian birds.

Another reason mtDNA has featured prominently in phylogeographic studies is that the data are relatively easy to obtain from traditional museum specimens (Payne & Sorenson, 2003). Inclusion of these “historical” samples improves geographic coverage over the reliance solely on newly collected specimens. However, nowadays historical specimens can also provide comprehensive genomic data (Bi et al., 2013). The acquisition of data from historical specimens requires that such specimens exist for given populations and yield DNA of adequate quality, which is often not the case. Historical specimens of birds from Southeast Asia generally represent only a small portion of a species' distributions, are old, and poorly documented, and their DNA is often severely degraded and may require substantial analytical modification (Lim & Braun, 2016). Nevertheless, the extraction of genome‐scale data from traditional specimens may permit more extensive phylogeographic inference for some species in the region.

**Figure 1:**
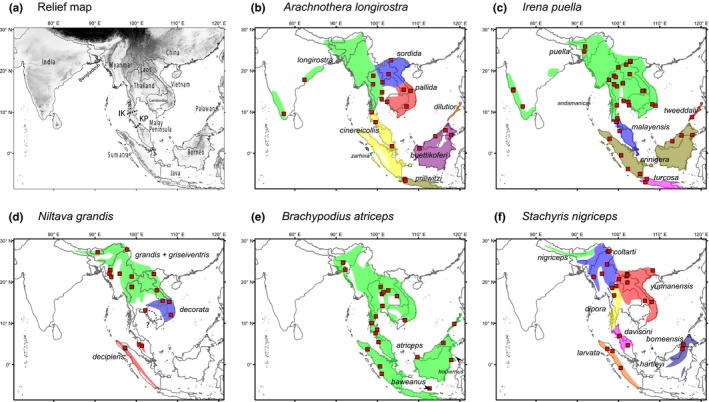
Relief map of the study region (darker = high elevation) and names of various geographic places (a). Localities of samples used (red boxes) and distributions of the five study species (b–f). For each study species, subspecies ranges are delineated based mainly on textual descriptions in Dickinson and Christidis (2014) and information from museum collections and are therefore approximate. Not all subspecies are shown, especially those found in small islands. A dashed line indicates uncertainty with regard to the identity of the subspecies that occupies that region. IK, Isthmus of Kra; KP, Kangar‐Pattani Line

Here, we present a phylogeographic comparison of five bird species codistributed across two Southeast Asian biogeographic subregions: Indochina, that is, easternmost India, Myanmar, Thailand, Cambodia, Laos, Vietnam, and westernmost China; and Sundaland, the Sunda continental shelf and its constituent lands, including the Malay Peninsula, Borneo, Sumatra, Java, and Palawan (Figure 1). The five species, representing five passerine families, are little spiderhunter *Arachnothera longirostra* (Nectariniidae), Asian fairy‐bluebird *Irena puella* (Irenidae), black‐headed bulbul *Brachypodius atriceps* (Pycnonotidae), large niltava *Niltava grandis* (Muscicapidae), and gray‐throated babbler *Stachyris nigriceps* (Timaliidae). These species were selected because they (a) are widespread and largely codistributed in continental Southeast Asia, and are usually considered single species, as opposed to groups comprising allospecies (the Palawan population of *Irena *is sometimes an exception); (b) are common and thus well represented in collections, a necessity for historical sampling; and (c) represent distinct ecological types and thus vary in dispersal potential and potentially genetic differentiation across space. *Arachnothera longirostra*, *I. puella*, and *B. atriceps* are eurytopic nectarivorous/insectivorous or frugivorous/insectivorous species that range widely among habitats and in elevation (Sheldon, Moyle, & Kennard, 2001; Wells, 2007). *Stachyris nigriceps* and *N. grandis* are insectivores inhabiting hill and submontane forest. As such, their dispersal potential is expected to be more habitat‐restricted than the first three species, a feature that has been linked with greater geographic structuring (Burney & Brumfield, 2009; Chua et al., 2017). Examination of intraspecific diversity in these five species provides a suite of examples of how species with varied ecologies have responded to the sea‐level and habitat changes that have occurred in Southeast Asia during the cyclic global glaciation events of the Pleistocene (Sheldon et al., 2015; Woodruff & Turner, 2009).

We assayed the genetic diversity of the study species using DNA sequences linked to thousands of ultraconserved elements (UCEs; Faircloth et al., 2012). This approach has proven useful for generating data from historical as well as modern specimens (Lim & Braun, 2016; McCormack, Tsai, & Faircloth, 2016; Ruane & Austin, 2017) and for both phylogenetic (Oliveros, Andersen, et al., 2019; Oliveros, Field, et al., 2019) and population‐level studies (Harvey, Aleixo, Ribas, & Brumfield, 2017; Smith, Harvey, Faircloth, Glenn, & Brumfield, 2014). We used these data to estimate population genetic structure in each species as well as population histories including divergence times and the incidence of past gene flow between populations. Our primary goals were (a) to evaluate the degree to which patterns of differentiation are concordant or discordant across species with diverse ecologies, and (b) to assess whether population genetic structure or demographic events were associated with landscape features or ecological traits of hypothesized evolutionary importance. These include: (a) the potential barrier represented by the Isthmus of Kra between Indochina and Sundaland (Hughes et al., 2003; Woodruff, 2003; Woodruff & Turner, 2009), (b) the biogeographic disjunction resulting in an east–west divide in central Indochina (Fuchs et al., 2015; Manawatthana et al., 2017; Reddy & Moyle, 2011), (c) the repeated island isolation and connection in Sundaland in the Pleistocene (Lim et al., 2017; Sheldon et al., 2015), and (d) the habitat requirements and corresponding dispersal potential of individual species (Chua et al., 2017; Lim et al., 2017). We discuss the potential importance of these drivers of evolution in Southeast Asian and identify novel patterns and processes in need of further study.

## 2 METHODS

### 2.1 Sampling, laboratory work, and data generation

Details on sample collection, molecular laboratory work, and generation of DNA sequence data were described in Lim and Braun (2016). Briefly, we obtained tissue samples (through museum loans or collecting) of 194 individuals belonging to the five study species (*Arachnothera longirostra*, *Irena puella*, *Brachypodius atriceps*, *Niltava grandis*, and *Stachyris nigriceps*) and nine outgroup taxa (Table 1). To obtain an appropriate distribution of specimens for comparison, we partitioned the study region into 28 subregions and attempted to obtain, given availability of existing specimens, an even sampling across them (Figure S1). These areas were delineated based largely on published bird distributions or areas of endemism (King, Woodcock, & Dickinson, 1975; Reddy, 2005; Robson, 2005). DNA was extracted from toe pads of historical specimens using phenol and chloroform or from fresh blood or tissue samples using Qiagen DNeasy Blood and Tissue Kits or an Autogen Gene Prep machine. DNA extracts were ligated with dual‐indexed Illumina adapters and enriched for ultraconserved element (UCE) loci using the 5472 120‐mer myBaits tetrapod capture probe set from MYcroarray, Inc., now Arbor Biosciences (Faircloth et al., 2012). For historical samples, DNA extraction and library preparation were conducted in the Smithsonian Institution's Center for Conservation Genomics ancient DNA laboratory. Each batch of historical DNA extractions consisted of up to eleven samples (each study species was represented by 2–3 toe‐pad samples) and one negative control. Each extraction control was tested for contamination with PCR using bird‐specific cytochrome *b* primers targeting a 307 bp region (Paxinos et al., 2002), and no contamination was detected in any of the controls. The enriched libraries were sequenced in three lanes of an Illumina HiSeq 2000 machine (performed by BGI Americas, Inc.) to generate 100 bp paired‐end reads. Of these samples, 20.1% did not produce sufficient sequence data (i.e., <*c*. 200,000 reads) to be used for downstream analyses. These failures were caused by poor template quality, failed library preparation, or unsuccessful target enrichment. Our final dataset contained high‐quality data for the 28 *A. longirostra*, 41 *I. puella*, 25 *N. grandis*, 30 *P. atriceps*, and 31 *S. nigriceps* (and up to two outgroup samples per species; Table 1). Of the successfully sequenced ingroup samples, 78.1% were historical, that is, from toe pads obtained from bird study skins that were collected between 1873 and 1986. The remaining samples were derived from muscle or blood collected fresh in the field (either frozen or in preservative) and archived in museum genetic resource collections.

**Table 1:** Samples (including outgroup taxa) used in this study and their collecting localities

Genus	Species	Institutional source	Year collected	Catalog number	Latitude	Longitude	Region	Cluster	Label
*Arachnothera*	*longirostra*	NMNH	1983	585214	17.83	82.33	India	India	1
*Arachnothera*	*longirostra*	NMNH	1986	585629	9.58	77.32	India	India	2
*Arachnothera*	*longirostra*	YPM	1957	47454	7.58	99.58	Tanintharyi	Indochina	3
*Arachnothera*	*longirostra*	FMNH	1931	91634	15.43	106.38	S Laos	Indochina	4
*Arachnothera*	*longirostra*	LSUMNS	Modern	52076	1.72	103.5	Malay Peninsula	Indochina	5
*Arachnothera*	*longirostra*	NMNH	1925	307418	13.13	101.04	E Thailand	Indochina	6
*Arachnothera*	*longirostra*	FMNH	1931	91636	15.43	106.38	S Laos	Indochina	7
*Arachnothera*	*longirostra*	FMNH	1929	80201	22.54	103.29	Tonkin Guangxi	Indochina	8
*Arachnothera*	*longirostra*	YPM	1959	68967	16.75	98.94	N Thailand	Indochina	9
*Arachnothera*	*longirostra*	NMNH	1930	307417	12.47	102.39	E Thailand	Indochina	10
*Arachnothera*	*longirostra*	NMNH	1965	534528	14.78	101.12	Central Thailand	Indochina	11
*Arachnothera*	*longirostra*	YPM	1938	18051	19.2	102.72	NC Laos	Indochina	12
*Arachnothera*	*longirostra*	YPM	1957	65321	18.8	99	N Thailand	Indochina	13
*Arachnothera*	*longirostra*	AMNH	Modern	12308	15.18	108.03	Annam	Indochina	14
*Arachnothera*	*longirostra*	YPM	1957	47450	9.95	98.61	Tanintharyi	Indochina	15
*Arachnothera*	*longirostra*	AMNH	Modern	10813	15.18	108.03	Annam	Indochina	16
*Arachnothera*	*longirostra*	KUNHM	Modern	23173	11.38	107.06	Annam	Indochina	17
*Arachnothera*	*longirostra*	KUNHM	Modern	23171	11.38	107.06	Annam	Indochina	18
*Arachnothera*	*longirostra*	YPM	1959	68966	16.75	98.94	N Thailand	Indochina	19
*Arachnothera*	*longirostra*	NMNH	1965	534529	14.78	101.12	Central Thailand	Indochina	20
*Arachnothera*	*longirostra*	NMNH	1954	459990	17.21	101.21	NE Thailand	Indochina	21
*Arachnothera*	*longirostra*	LSUMNS	Modern	58174	4.16	114.03	Brunei Sarawak	Borneo	22
*Arachnothera*	*longirostra*	LSUMNS	Modern	52126	1.18	110.2	Brunei Sarawak	Borneo	23
*Arachnothera*	*longirostra*	LSUMNS	Modern	38541	4.4	117.89	Sabah	Borneo	24
*Arachnothera*	*longirostra*	LSUMNS	Modern	36446	5.58	116.49	Sabah	Borneo	25
*Arachnothera*	*longirostra*	NMNH	1909	219072	−6.6	106.8	Java	Java	26
*Arachnothera*	*longirostra*	NMNH	1909	220091	−6.21	106.61	Java	Java	27
*Arachnothera*	*longirostra*	NMNH	1909	219071	−6.6	106.8	Java	Java	28
*Arachnothera*	*crassirostris*	KUNHM	Modern	24436			Sarawak		
*Arachnothera*	*robusta*	LSUMNS	Modern	36483			Sabah		
*Irena*	*puella*	NMNH	1954	452227	16.99	101.08	Central Thailand	IC + MP	1
*Irena*	*puella*	YPM	1949	9680	25.9	91.9	Himalayan foothills	IC + MP	2
*Irena*	*puella*	NMNH	1953	450549	16.26	99.61	Central Thailand	IC + MP	3
*Irena*	*puella*	YPM	1960	68529	16.75	98.94	N Thailand	IC + MP	4
*Irena*	*puella*	FMNH	1929	79464	22.37	102.85	Tonkin Guangxi	IC + MP	5
*Irena*	*puella*	YPM	1959	68535	16.75	98.94	N Thailand	IC + MP	6
*Irena*	*puella*	YPM	1958	76836	24.7	91.68	Himalayan foothills	IC + MP	7
*Irena*	*puella*	NMNH	1928	311146	12.47	102.39	E Thailand	IC + MP	8
*Irena*	*puella*	ANSP	1935	124243	12.68	101.24	E Thailand	IC + MP	9
*Irena*	*puella*	FMNH	1929	79454	22.21	102.91	Tonkin Guangxi	IC + MP	10
*Irena*	*puella*	ANSP	1933	114178	18.49	99.3	N Thailand	IC + MP	11
*Irena*	*puella*	ANSP	1938	131513	7.65	99.45	Tanintharyi	IC + MP	12
*Irena*	*puella*	NMNH	1965	534777	8.49	99.73	Tanintharyi	IC + MP	13
*Irena*	*puella*	ANSP	1928	82751	18.82	98.89	N Thailand	IC + MP	14
*Irena*	*puella*	FMNH	1938	237499	15.27	74.51	W Ghats	India	15
*Irena*	*puella*	ANSP	1937	130338	11.64	99.59	Central Thailand	IC + MP	16
*Irena*	*puella*	AMNH	1938	462684	15.47	74.52	W Ghats	India	17
*Irena*	*puella*	YPM	1938	17353	19.2	102.72	NC Laos	IC + MP	18
*Irena*	*puella*	ANSP	1934	123311	17	101	Central Thailand	IC + MP	19
*Irena*	*puella*	NMNH	1961	475601	11.88	108.2	Annam	IC + MP	20
*Irena*	*puella*	FMNH	1931	90856	15.11	105.8	S Laos	IC + MP	21
*Irena*	*puella*	ANSP	1933	114180	20.87	99.94	Shan	IC + MP	22
*Irena*	*puella*	AMNH	1890	565002	17.82	97.68	E Myanmar	IC + MP	23
*Irena*	*puella*	YPM	?	47008	7.58	99.58	Tanintharyi	IC + MP	24
*Irena*	*puella*	NMNH	1918	278423	11.56	108.99	Annam	IC + MP	25
*Irena*	*puella*	AMNH	1873	565014	11.35	76.8	W Ghats	India	26
*Irena*	*puella*	FMNH	1931	90853	15.18	106.24	S Laos	IC + MP	27
*Irena*	*puella*	FMNH	1929	79475	21.51	101.85	NC Laos	IC + MP	28
*Irena*	*puella*	AMNH	1891	565003	17.82	97.68	E Myanmar	IC + MP	29
*Irena*	*puella*	NMNH	1914	249000	11.66	102.56	E Thailand	IC + MP	30
*Irena*	*puella*	ANSP	1901	38966	−0.46	100.61	West Sumatra	Sum + Bor	31
*Irena*	*puella*	ANSP	1939	139744	3.51	97.82	E Sumatra	Sum + Bor	32
*Irena*	*puella*	MVZ	1923	43865	5.43	100.27	Malay Peninsula	IC + MP	33
*Irena*	*puella*	MCZ	1936	177788	−3.8	102.27	West Sumatra	Sum + Bor	34
*Irena*	*puella*	LSUMNS	Modern	51044	4.4	117.89	Sabah	Sum + Bor	35
*Irena*	*puella*	ANSP	1901	39172	−4.99	105.21	E Sumatra	Sum + Bor	36
*Irena*	*puella*	ANSP	?	56492	−7	106.5	Java	Sum + Bor	37
*Irena*	*puella*	FMNH	1939	213980	−6.21	106.85	Java	Sum + Bor	38
*Irena*	*puella*	YPM	Modern	142997	4.34	115.26	Brunei Sarawak	Sum + Bor	39
*Irena*	*puella*	LSUMNS	Modern	57075	2.94	113.03	Brunei Sarawak	Sum + Bor	40
*Irena*	*puella*	YPM	1962	73857	8.78	117.83	Palawan	Palawan	41
*Irena*	*cyanogastra*	KUNHM	Modern	14294			Leyte		
*Chloropsis*	*venusta*	LSUMNS	Modern	70063			Sumatra		
*Niltava*	*grandis*	NMNH	1966	535501	13.1	102.19	E Thailand	E Thailand	1
*Niltava*	*grandis*	NMNH	1966	535506	13.1	102.19	E Thailand	E Thailand	2
*Niltava*	*grandis*	MVZ	1970	160375	4.52	101.38	Malay Peninsula	Sum + MP	3
*Niltava*	*grandis*	MVZ	1923	44126	4.85	100.73	Malay Peninsula	Sum + MP	4
*Niltava*	*grandis*	YPM	1938	41013	4.07	97.23	E Sumatra	Sum + MP	5
*Niltava*	*grandis*	ANSP	1939	139566	3.92	97.35	E Sumatra	Sum + MP	6
*Niltava*	*grandis*	ANSP	1939	139563	3.92	97.35	E Sumatra	Sum + MP	7
*Niltava*	*grandis*	NMNH	1939	359147	12	108.4	Annam	Indochina	8
*Niltava*	*grandis*	FMNH	1931	91439	18	105	NC Laos	Indochina	9
*Niltava*	*grandis*	FMNH	1931	91441	15.43	106.38	S Laos	Indochina	10
*Niltava*	*grandis*	AMNH	Modern	12300	15.18	108.03	Annam	Indochina	11
*Niltava*	*grandis*	ANSP	1933	112990	18.83	98.89	N Thailand	Indochina	12
*Niltava*	*grandis*	KUNHM	Modern	27995	21.95	104.26	Tonkin Guangxi	Indochina	13
*Niltava*	*grandis*	AMNH	1938	306183	21.23	93.92	Chin Rakhine	Indochina	14
*Niltava*	*grandis*	ANSP	1933	112994	21.32	98.9	Shan	Indochina	15
*Niltava*	*grandis*	NMNH	Modern	620545	22.9	93.7	Chin Rakhine	Indochina	16
*Niltava*	*grandis*	KUNHM	Modern	27994	21.95	104.26	Tonkin Guangxi	Indochina	17
*Niltava*	*grandis*	NMNH	1967	519870	27.23	90.65	Himalayan foothills	Indochina	18
*Niltava*	*grandis*	KUNHM	Modern	15257	22	96	Chin Rakhine	Indochina	19
*Niltava*	*grandis*	ANSP	1933	114777	18.79	98.96	N Thailand	Indochina	20
*Niltava*	*grandis*	ANSP	1928	87134	18.82	98.89	N Thailand	Indochina	21
*Niltava*	*grandis*	NMNH	Modern	631832	27.83	97.76	Kachin Sagaing	Indochina	22
*Niltava*	*grandis*	ANSP	1938	137713	22	93.5	C Myanmar	Indochina	23
*Niltava*	*grandis*	AMNH	1938	306186	21.23	93.92	Chin Rakhine	Indochina	24
*Niltava*	*grandis*	ANSP	1938	137711	22	93.5	Chin Rakhine	Indochina	25
*Niltava*	*macgregoriae*	KUNHM	Modern	10341			Guangxi		
*Niltava*	*davidi*	KUNHM	Modern	11093			Guizhou		
*Brachypodius*	*atriceps*	ANSP	1932	112576	14.2	101.23	E Thailand	E Indochina	1
*Brachypodius*	*atriceps*	ANSP	1933	112577	8.43	99.96	Tanintharyi	W IC + Sunda	2
*Brachypodius*	*atriceps*	BURKE	Modern	116982	−2.27	101.03	West Sumatra	W IC + Sunda	3
*Brachypodius*	*atriceps*	KUNHM	Modern	12641	9.84	118.64	Palawan	W IC + Sunda	4
*Brachypodius*	*atriceps*	KUNHM	Modern	12642	9.84	118.64	Palawan	W IC + Sunda	5
*Brachypodius*	*atriceps*	ANSP	1939	139756	3.69	97.6	E Sumatra	W IC + Sunda	6
*Brachypodius*	*atriceps*	ANSP	1939	139759	3.69	97.6	E Sumatra	W IC + Sunda	7
*Brachypodius*	*atriceps*	MVZ	1965	156056	10.75	106.67	Annam	E Indochina	8
*Brachypodius*	*atriceps*	MVZ	1965	156057	10.75	106.67	Annam	E Indochina	9
*Brachypodius*	*atriceps*	YPM	1945	17185	16.57	104.75	S Laos	E Indochina	10
*Brachypodius*	*atriceps*	YPM	1944	17186	16.57	104.75	S Laos	E Indochina	11
*Brachypodius*	*atriceps*	NMNH	1907	181552	−5.8	112.65	Java	W IC + Sunda	12
*Brachypodius*	*atriceps*	NMNH	1913	182876	1.1	117.9	Kalimantan Timur	W IC + Sunda	13
*Brachypodius*	*atriceps*	NMNH	1929	313386	17.96	102.61	NC Laos	E Indochina	14
*Brachypodius*	*atriceps*	NMNH	1929	313387	17.96	102.61	NC Laos	E Indochina	15
*Brachypodius*	*atriceps*	ANSP	1901	38984	−0.46	100.61	West Sumatra	W IC + Sunda	16
*Brachypodius*	*atriceps*	MVZ	1921	42709	5.43	100.27	Malay Peninsula	W IC + Sunda	17
*Brachypodius*	*atriceps*	MVZ	1923	43852	5.43	100.27	Malay Peninsula	W IC + Sunda	18
*Brachypodius*	*atriceps*	NMNH	1952	450852	11.63	99.6	Central Thailand	W IC + Sunda	19
*Brachypodius*	*atriceps*	NMNH	1955	459653	17.48	101.5	NE Thailand	E Indochina	20
*Brachypodius*	*atriceps*	NMNH	1955	459657	17.06	101.09	Central Thailand	E Indochina	21
*Brachypodius*	*atriceps*	YPM	?	47011	7.58	99.58	Tanintharyi	W IC + Sunda	22
*Brachypodius*	*atriceps*	YPM	1957	47021	10	98.6	Tanintharyi	W IC + Sunda	23
*Brachypodius*	*atriceps*	LSUMNS	Modern	58540	1.8	109.71	Brunei Sarawak	W IC + Sunda	24
*Brachypodius*	*atriceps*	YPM	?	64313	18.8	100.8	N Thailand	E Indochina	25
*Brachypodius*	*atriceps*	YPM	?	64314	18.8	100.8	N Thailand	W IC + Sunda	26
*Brachypodius*	*atriceps*	YPM	1958	77504	23	92.25	Himalayan foothills	W IC + Sunda	27
*Brachypodius*	*atriceps*	YPM	1958	77511	24.7	91.68	Himalayan foothills	W IC + Sunda	28
*Brachypodius*	*atriceps*	ANSP	Modern	16118	5.23	116	Sabah	W IC + Sunda	29
*Brachypodius*	*atriceps*	ANSP	Modern	16185	5.23	116	Sabah	W IC + Sunda	30
*Euptilotus*	*eutilotus*	LSUMNS	Modern	57023			Sarawak		
*Stachyris*	*nigriceps*	AMNH	Modern	10737	15.18	108.03	Annam	E Indochina	1
*Stachyris*	*nigriceps*	LSUMNS	Modern	78736	3.78	115.48	Brunei Sarawak	Borneo	2
*Stachyris*	*nigriceps*	LSUMNS	Modern	78756	3.78	115.48	Brunei Sarawak	Borneo	3
*Stachyris*	*nigriceps*	KUNHM	Modern	15245	22	96	C Myanmar	W Indochina	4
*Stachyris*	*nigriceps*	KUNHM	Modern	15246	22	96	C Myanmar	W Indochina	5
*Stachyris*	*nigriceps*	MCZ	1938	265882	21.23	93.92	Chin Rakhine	W Indochina	6
*Stachyris*	*nigriceps*	MCZ	1938	265883	21.23	93.92	Chin Rakhine	W Indochina	7
*Stachyris*	*nigriceps*	ANSP	1939	139856	3.81	97.28	E Sumatra	Sum + MP	8
*Stachyris*	*nigriceps*	AMNH	1918	589692	3.27	98.55	E Sumatra	Sum + MP	9
*Stachyris*	*nigriceps*	NMNH	Modern	15183	27.5	97.8	Kachin Sagaing	W Indochina	10
*Stachyris*	*nigriceps*	YPM	1959	38021	27.37	97.45	Kachin Sagaing	W Indochina	11
*Stachyris*	*nigriceps*	AMNH	1908	589674	24.27	97.23	Kachin Sagaing	W Indochina	12
*Stachyris*	*nigriceps*	AMNH	1901	589678	4.63	102.24	Malay Peninsula	Sum + MP	13
*Stachyris*	*nigriceps*	AMNH	1901	589679	4.63	102.24	Malay Peninsula	Sum + MP	14
*Stachyris*	*nigriceps*	MCZ	1937	196421	18.58	98.48	N Thailand	E Indochina	15
*Stachyris*	*nigriceps*	MCZ	1937	196424	18.58	98.48	N Thailand	E Indochina	16
*Stachyris*	*nigriceps*	NMNH	1930	330586	19	99.42	N Thailand	E Indochina	17
*Stachyris*	*nigriceps*	YPM	1959	68729	16.75	98.94	N Thailand	E Indochina	18
*Stachyris*	*nigriceps*	MCZ	1939	267896	19.88	102.14	NC Laos	E Indochina	19
*Stachyris*	*nigriceps*	MCZ	1939	267907	21.92	102.1	NC Laos	E Indochina	20
*Stachyris*	*nigriceps*	FMNH	1929	78922	21.68	102.1	NC Laos	E Indochina	21
*Stachyris*	*nigriceps*	FMNH	1929	78924	21.39	101.97	NC Laos	E Indochina	22
*Stachyris*	*nigriceps*	FMNH	1931	91010	15.43	106.38	S Laos	E Indochina	23
*Stachyris*	*nigriceps*	FMNH	1931	91030	15.43	106.38	S Laos	E Indochina	24
*Stachyris*	*nigriceps*	LSUMNS	Modern	51000	4.85	115.7	Sabah	Borneo	25
*Stachyris*	*nigriceps*	LSUMNS	Modern	51017	4.85	115.7	Sabah	Borneo	26
*Stachyris*	*nigriceps*	ANSP	1933	112341	20.7	100.11	Shan	E Indochina	27
*Stachyris*	*nigriceps*	ANSP	1933	112342	20.7	100.11	Shan	E Indochina	28
*Stachyris*	*nigriceps*	NMNH	1964	534904	6.94	100.26	Tanintharyi	Sum + MP	29
*Stachyris*	*nigriceps*	KUNHM	Modern	9957	22.8	108.3	Tonkin Guangxi	E Indochina	30
*Stachyris*	*nigriceps*	BURKE	Modern	117024	−0.84	100.53	West Sumatra	Sum + MP	31

Note

**Figure 2:**
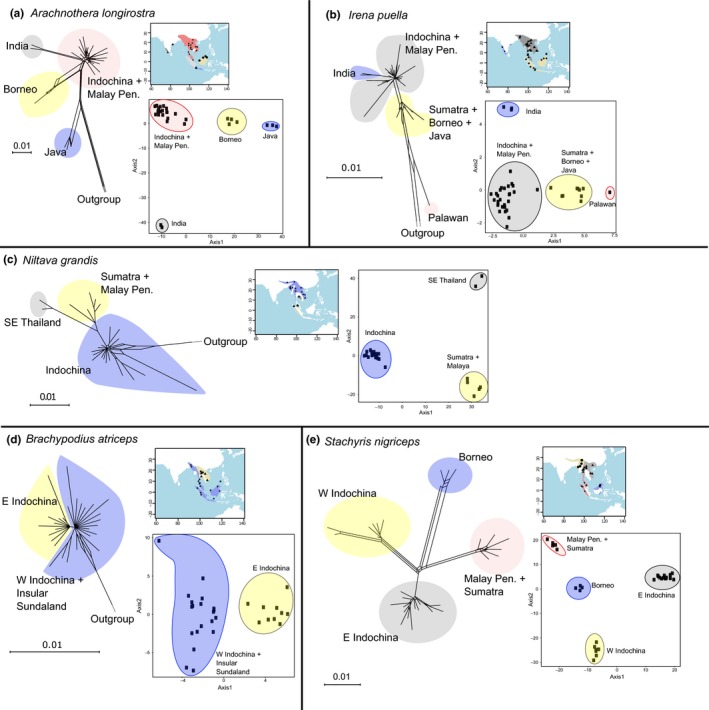
Phylogenetic network and PCA plot for each of the study species (a–e). In each phylogenetic network, the color of each cluster of individuals corresponds to the color in the inset map

Modern genetic samples (preserved frozen tissues or blood) are labeled as “modern” under year collected. If the date of collection is unknown, it is labeled as “?”. Region = sampling region indicated in Figure S1. Cluster = genetic cluster defined in Figure 2. Labels of samples match those found in tip labels of phylogenetic networks in Figure 2.

Abbreviations: Bor, Borneo; IC, Indochina; MP, Malay Peninsula; Sum, Sumatra.

### 2.2 Bioinformatics processing and data analysis

The bioinformatics workflow to generate genotype data for each species group was to: (a) build a pseudoreference genome for each species using contigs from a subset of individuals (10–15 individuals per species) using Phyluce version 1 and Geneious version 7.0.6 (Faircloth, 2016); (b) map reads of all individuals of a species group to the reference genome using Bowtie version 2 (Langmead & Salzberg, 2012); (c) conduct read deduplication with PicardTools version 1.122; (d) conduct probabilistic single nucleotide polymorphism (SNP) and genotype calling with GATK version 3.2 (DePristo et al., 2011); and (e) filter for high‐quality SNPs and genotypes. For SNP and genotype filtering, we followed general recommendations in GATK best practice documents and other publications (e.g., Carson et al., 2014). We used GATK's VariantFiltration tool to conduct both SNP (filter expression: ReadPosRankSum <−1.96 || ReadPosRankSum > 1.96 || BaseQRankSum <−1.96 || BaseQRankSum > 1.96 || MQRankSum <−1.96 || MQRankSum > 1.96 || FS > 20.0 || MQ < 30.0) and genotype filtering (filter expression: GQ < 13 || DP < 8) (Lim & Braun, 2016). Using mapDamage version 2.0, we observed characteristic postmortem damage in the DNA of historical samples, which includes higher rates of C →T and G →A misincorporations at the 5′ and 3′ ends of reads, respectively, due to increased deamination of C residues along single‐stranded overhangs (Jonsson, Ginolhac, Schubert, Johnson, & Orlando, 2013; Parks & Lambert, 2015). In addition to stringent SNP and genotype filtering, we overcame these issues using soft‐clipping. Specifically, we used very sensitive local alignment that allowed for soft‐clipping of ends of reads during Bowtie read mapping (see details in Lim & Braun, 2016). This option removed most of the C → T or G → A errors because they tend to occur near the beginning of each read (Gilbert et al., 2003). Finally, we output data in variant call format (VCF) files that were then subset and parsed to conduct a variety of downstream analyses.

### 2.3 Phylogenetic network of concatenated SNPs

We converted the VCF file for each species into a multisequence alignment file in fasta format (length: 1,466–10,062 bp), concatenating all the SNPs using PGDSpider version 2.1 (Lischer & Excoffier, 2012). For each individual, heterozygous sites were collapsed into IUPAC ambiguity codes and indel variants were ignored (i.e., not converted into sequence data). We then trimmed the data to generate data matrices that were 80% full (i.e., no alignment columns had more than 20% unknown bases, N) using a custom script (prune_Q_pub.py, see Data Availability). Next, we used jmodeltest version 2.1.3 to find the best substitution model for each alignment using these options: five substitution schemes, unequal base frequencies and no rate variation among sites, and tree search strategy = best (Posada, 2008). The best models were selected based on the Bayesian information criterion (BIC). Following this step, we used the jmodeltest substitution models and sequence alignments in SplitsTree version 4.12.6 to calculate pairwise genetic distances, which were in turn used to generate phylogenetic networks with the NeighborNet algorithm (Huson & Bryant, 2006). Each NeighborNet network is a collection of splits, with each split representing one bipartition of the taxa. To simplify the networks and reduce the number of branches, we filtered splits by removing any split whose weight fell below a given threshold (5 × 10^−4^, Table 2).

**Table 2:** Statistics related to sample sizes, amount of data generated per species, and population genetic analyses

	*Arachnothera longirostra*	*Irena puella*	*Niltava grandis*	*Brachypodius atriceps*	*Stachyris nigriceps*
Total no. of samples	28	41	25	30	31
No. of outgroup samples	2	2	2	1	0
Average length of UCE loci (bp)	432.8	348	719.5	346	705.1
No. of UCE loci with => 1 SNP	1,726	753	4,051	2,432	3,506
Total no. of SNPs	5,890	3,919	18,472	11,626	9,154
Number of SNPs that have no more than 50% missing data	5,886	3,864	18,467	11,624	9,154
Average number of called SNP genotypes per individual (range)	4,176.1 (1,632–5,864)	3,310.8 (1,408–3,889)	14,707.9 (5,390–18,389)	9,459.5 (4,293–11,503)	7,536.8 (3,655–9,119)
Phylogenetic network analysis					
No. of SNPs used in phylogenetic network	1,466	2,631	10,062	6,959	5,660
Substitution model used when constructing network	HKY, ti/tv = 3.0674	GTR, rmat = (0.7945 6.9869 2.0250 0.6547 6.7655)	HKY, ti/tv = 3.0301	GTR, rmat = (1.0023 7.3483 1.9447 0.7867 7.7714)	GTR, rmat = (1.1373 7.0933 1.8054 0.7654 8.7086)
No. of splits retained after/before filtering	56/78	49/106	43/71	53/92	58/101
Proportional weight retained after filtering	98.5%	97.9%	98.9%	98.9%	95.7%
PCA, DAPC					
No. of independent SNPs used for PCA and structure analysis	1,461	714	3,618	2,336	3,499
Proportional variance explained by PC 1	0.347	0.330	0.288	0.147	0.272
Proportional variance explained by PC 2	0.190	0.080	0.127	0.093	0.236
Number of clusters in DAPC	4	4	3	2	4
Structure analysis					
Average proportion of nonmissing SNP data per individual	68.7%	81.1%	76.2%	78.9%	84.4%
Optimal *K* in Structure analysis	5	3	2	3	5
*K* of additional Structure analysis results (Figure S3)	4,6	2,4	3	2,4	4,6

### 2.4 PCA, DAPC, and genetic differentiation

For ingroup samples in each species, we randomly selected one high‐quality biallelic SNP (i.e., passing SNP filters shown in data analysis) from each UCE locus using dplyr (https://dplyr.tidyverse.org) in R (R Development Core Team, 2014). This generated data matrices with 714–3,499 independent SNPs from each species (termed the independent‐SNPs dataset).

Using these SNP datasets, we conducted principal component analysis (PCA), followed by discriminant analysis of principal components (DAPC; Jombart, Devillard, & Balloux, 2010) for each species using the R packages adegenet version 2.0.0 and ade4 (Dray & Dufour, 2007; Jombart & Ahmed, 2011). Because principal component methods generally require complete datasets, we imputed missing values using the function impute PCA in the R package MissMDA (Josse & Husson, 2016). PCA was then performed with ade4 package function dudi.pca (center = T, scale = F). We conducted DAPC based on group memberships that were predefined using a series of complementary strategies. These strategies included inferring groups based on: SplitsTree phylogenetic networks, a successive *K*‐means method (find.cluster function in adegenet), locations of individuals on PCA plots, and geographic proximity among samples. Prior to running DAPC, we first performed stratified cross‐validation (*n* = 100 repetitions) to determine an optimal number of principal components to use for each analysis, which minimized the risk of model overfitting. Using the R package hierfstat version 0.04.22 (de Meeus & Goudet, 2007), we also calculated for each species the overall F_ST_, between‐group F_ST_ values, and Nei's genetic distances (Nei, 1972; Weir & Cockerham, 1984).

### 2.5 Structure analysis

To simultaneously identify genetic cluster membership and genetic admixture of individuals, we analyzed the independent‐SNPs data with the MCMC‐based program Structure version 2.3.4 (Pritchard, Stephens, & Donnelly, 2000). For each species, we ran Structure using the admixture model with correlated allele frequencies and by specifying *K* (number of genetic clusters) from 1 to 8. For each *K* value, we conducted 10 independent runs, setting burn‐in and number of MCMC steps to 100,000 each.

We determined an optimal *K* value for each species using the Δ*K* method described by Evanno, Regnaut, and Goudet (2005), which was implemented in the Structure Harvester version 0.6.94 web server (Earl & vonHoldt, 2012). Because of complexities in real‐world population structure (e.g., hierarchical structure) and assumptions made by Structure's admixture model that may be violated (e.g., no clinal genetic variation, absence of an unsampled source population, lack of a strong bottleneck, all populations well sampled), we also present Structure results based on additional *K* values (optimal *K* ± 1) and focused on interpreting aspects of its outputs that are corroborated by other analyses and the geographic locations of individuals, while keeping in mind the limitations of Structure analyses (Gao, Bryc, & Bustamante, 2011; Lawson, van Dorp, & Falush, 2018; Porras‐Hurtado et al., 2013). For each *K* value, we used CLUMPP version 1.1.2 and the Greedy search option to obtain cluster membership coefficients for each individual averaged across the 10 runs (Jakobsson & Rosenberg, 2007).

### 2.6 Population branching and delimitation analyses

We ran SNAPP species tree analysis in BEAST version 2.3.3 to infer branching patterns of populations in each species using the independent‐SNPs datasets and populations delineated in DAPC analyses (Bryant, Bouckaert, Felsenstein, Rosenberg, & RoyChoudhury, 2012). We ran two analyses for each of the five study species. One of the analyses included only ingroup samples; the other had an outgroup taxon included (except for *Stachyris*, for which we lacked outgroup data). We used the resulting topologies to guide population lumping in BFD* analyses and tree building for G‐PhoCS analyses (see below). We ran each SNAPP analysis for at least one million generations, sampling every 1,000 generations. The first 10% of the MCMC chains were discarded as burn‐in, and log‐normal distributions were used for lambda and rate priors.

We conducted Bayes factor delimitation of populations using BFD* implemented in BEAST version 2.3.3 to evaluate support for alternative sample assignment schemes in which populations are increasingly lumped together (Bouckaert, 2014; Leache, Fujita, Minin, & Bouckaert, 2014). BFD* uses path sampling to estimate marginal likelihoods of different population assignment models (i.e., different ways of assigning individuals into populations). We started with the model with the largest number of populations (used in DAPC analyses) and progressively grouped these populations together based on the branching pattern determined by SNAPP analyses. Following this step, we compared the marginal likelihood estimate (MLE) of each of the alternative models against the best model and calculated a Bayes factor: BF = 2 × difference in MLE (Leache et al., 2014). For the BFD* analysis of each species, we used the independent‐SNPs datasets, but converted them into a binary (1's and 0's) format. To assess the rate at which marginal likelihoods stabilized, we ran each analysis for 12 or 24 path sampling steps (alpha parameter = 0.3, 50% burn‐in), but only report results from the latter. Each MCMC chain length was set to 200,000 generations (20,000 pre‐burn‐in generations), sampled every 1,000 generations. For priors, we used default settings and chose the log‐normal distribution for lambda and the Yule model birth rate prior (rates not sampled).

### 2.7 Estimation of demographic parameters and divergence time

To jointly estimate the demographic parameters related to the divergence history of populations in each species (i.e., population divergence time, population size, and gene flow rate), we generated species‐specific haplotype data for each UCE locus and analyzed each set of alignments with the coalescent‐based tool G‐PhoCS version 1.2.3 (Gronau, Hubisz, Gulko, Danko, & Siepel, 2011). To generate the haplotype data, we first ran the UnifiedGenotyper tool of GATK using the output mode EMIT_ALL_SITES. This produced VCF files for every nucleotide site, both variable and invariant. Using PGDSpider version 2.1, each VCF file was converted into a single fasta alignment file with unphased haplotype data of all UCE loci concatenated (two unphased haplotypes per individual). Next, we converted the haplotype data into a diplotype format (with IUPAC ambiguities, one diplotype per individual) using a custom script (2hap_into_diplotype.py, see Data Availability), split the constituent UCE loci into individual fasta alignment files, and trimmed them at the 5′ and 3′ ends to remove sites containing > 50% N (unknown nucleotide bases). Following this, we ran mstatspop on each alignment to generate population genetics summary statistics (https://github.com/CRAGENOMICA/mstatspop). After inspection of histograms and distribution plots of summary statistics, we filtered UCE loci based on the following removal criteria to minimize the chance of including alignments that were made up of low‐quality or nonhomologous reads: (a) alignments that were less than 200 bp in length and (b) alignments that had a high number of polymorphic sites per bp (i.e., the top 5%). With the filtered alignment data, we conducted another round of mstatspop analyses to calculate population genetics summary statistics and generated input data for G‐PhoCS.

For each species' G‐PhoCS analyses, the underlying guide tree (population divergence) topology was derived from the results of SNAPP analyses. We used population divergence models in which bidirectional gene flow between pairs of modern, geographically adjacent populations was allowed (migration model) and diffuse gamma priors were set for all demographic parameters. The shape (*α*) and scale (*β*) parameters that define gamma prior distributions for each of the demographic parameters were as follows: *α* = 1 and *β* = 5,000 for both *θ* and *τ*, and *α* = 1 and *β* = 0.01 for migration rates. For each species, we conducted some initial MCMC runs with auto‐fine‐tuning to evaluate run speed, convergence, and mixing. To produce the final results for each species, we combined data from 4 to 6 MCMC runs, each up to 100,000 iterations long. We used Tracer version 1.6 to evaluate marginal posterior distributions and trace plots of parameters and likelihood values (Rambaut, Suchard, Xie, & Drummond, 2014). The strategy of combining different MCMC runs to increase the effective sample size (ESS) of each estimated parameter was needed because the version of G‐PhoCS we used was single‐threaded and required weeks to months of run time to reach 100,000 MCMC iterations.

To obtain demographic parameters in absolute units (e.g., number of individuals and number of years) rather than in substitution‐rate‐scaled units, we estimated an approximate substitution rate for the UCE loci. To do this, we used the time‐calibrated phylogenetic tree of oscine songbirds by Moyle et al. (2016). This tree is based on data generated using the same UCE probe set (Mycroarray myBaits Tetrapod UCE 5K) and was calibrated using secondary calibration points and the date of the *Orthonyx kaldowinyeri* fossil. Their complete data matrix had a longer average alignment length (728 bp) than our G‐PhoCS datasets but contained fewer loci (515 UCE loci). We calculated pairwise divergence time distances between branch tips in the Moyle et al. (2016) tree using the cophenetic.phylo function of R package ape version 5 (Paradis & Schliep, 2018). We also calculated pairwise distances between individual concatenated UCE sequences using dist.dna and the substitution model TNY93 + GAMMA. We then estimated the average number of substitutions per myr and assumed the generation time of each species to be 1.7 years (Saether et al., 2007). This estimate was used to convert the following mutation‐rate‐scaled demographic parameters produced by G‐PhoCS: *θ* (= 4*N*_e_ *μ*, where *N*_e_ is the effective population size and *μ* the mutation rate per nucleotide site per generation); divergence time *τ* (= *Tμ*, where *T* is the divergence time in generations) between populations; and per‐generation migration rates between current populations *M*_ST_/*μ*, in which *M*_ST_ is the proportion of individuals in the receiving population *T* that immigrated from the source population *S* (Gronau et al., 2011). The point estimate of substitution rate resulting from this approach is approximate due to uncertainty in the branch length estimates in both our dataset and the calibration dataset.

## 3 RESULTS

We obtained an average of 6.88 million (*SD* = 4.86 million) quality‐trimmed and filtered reads per individual in our final dataset (*N* = 162). After SNP and genotype filtering, the total number of SNPs per species ranged from 3,919 to 18,472, and the number of variable UCE loci ranged from 753 to 4,051 (Table 2). The amount of missing data was low; when SNPs having more than 50% missing data (missing genotypes) were removed, the number of SNPs retained per species group spanned 3,864 to 18,467. Using mapDamage 2.0, we observed characteristic postmortem damage in the DNA of historical samples (Jonsson et al., 2013). However, we were able to significantly reduce its impact on genotype accuracy by trimming read ends during read mapping (local alignment, see Section 2). Our analyses showed that this approach reduced C →T or G →A substitutions near the beginning of each read from an initial 10% to 25% down to 1%–2%, a rate similar to that of other types of nucleotide substitutions in the untrimmed portions of the reads (Lim & Braun, 2016; Figure S9).

### 3.1 Phylogenetic networks

The number of SNPs used to construct phylogenetic networks ranged from 1,466 to 10,062. Network analysis identified two to four clusters of individuals in each species (Figure 2). The *Arachnothera longirostra* network contains a geographically central cluster of individuals from Indochina and the Malay Peninsula (Figure 2a). Two samples from India attach to this cluster but are separated by long branches. Bornean birds are the next most closely related group, followed by three individuals from Java. As with *A. longirostra*, the *Irena puella* network contains a large central cluster from Indochina and the Malay Peninsula; three individuals from India are nested within it (Figure 2b). Separated from this cluster are individuals from the Greater Sunda Islands. A single sample from Palawan is distantly related to all other *I. puella* individuals. In *Niltava grandis*, samples are separated into two main clusters. Most individuals from Indochina form a northern cluster, whereas birds from southeastern Thailand, the Malay Peninsula, and Sumatra form a more southerly cluster (Figure 2c). Within the northern cluster, five individuals from the eastern side of the region (nos. 8–13, Figure S2; Laos and Vietnam) are slightly differentiated from the rest, with individuals from southern Vietnam more differentiated. Instead of a north–south break, *Brachypodius atriceps* (Figure 2d) is divided into eastern (Laos, Vietnam, and Thailand) and western groups (western Myanmar and other parts of Southeast Asia). *Stachyris nigriceps*comprises four distinct clusters (Figure 2e). The two northern clusters are divided into eastern and western Indochinese groups, similar to *B. atriceps*. In the south are two clusters, one on Borneo and the other on the Malay Peninsula and Sumatra.

### 3.2 Population structure and differentiation

Principal components explain a substantial amount of population genetic variation in each species, with eigenvalues of the first two PCs accounting for 24.0% (*B. atriceps*) to 53.6% (*A. longirostra*) of the total variance (Table 2). Results of PCA (Figures 2 and S2) and DAPC (Figure S3) of population genetic structure correspond closely to the network analysis results. The four distinct PCA clusters in *A. longirostra* (Figure 2a) match those in the network, with Indian and Indochinese individuals segregating along axis 2. A similar pattern occurs in *I. puella* (Figure 2b), with Indian and Indochinese individuals differentiated along axis 2, whereas the other three populations separate along axis one. An individual from the Malay Peninsula (Table 1 and Figure S2, no. 33) occurs along axis one at a location intermediate between the Indochinese and the Borneo/Sumatra cluster. In *N. grandis* (Figure 2c), the three network clusters are represented in the PCA, with southeastern Thailand separated from Sumatra/Malay Peninsula individuals along axis 2. The population genetic structures of *B. atriceps* and *S. nigriceps* (Figure 2d,e, respectively) differ from the previous three species in that two genetic clusters in each were found within Indochina, separated from one another approximately at the boundary between Thailand and Myanmar.

**Figure 3:**
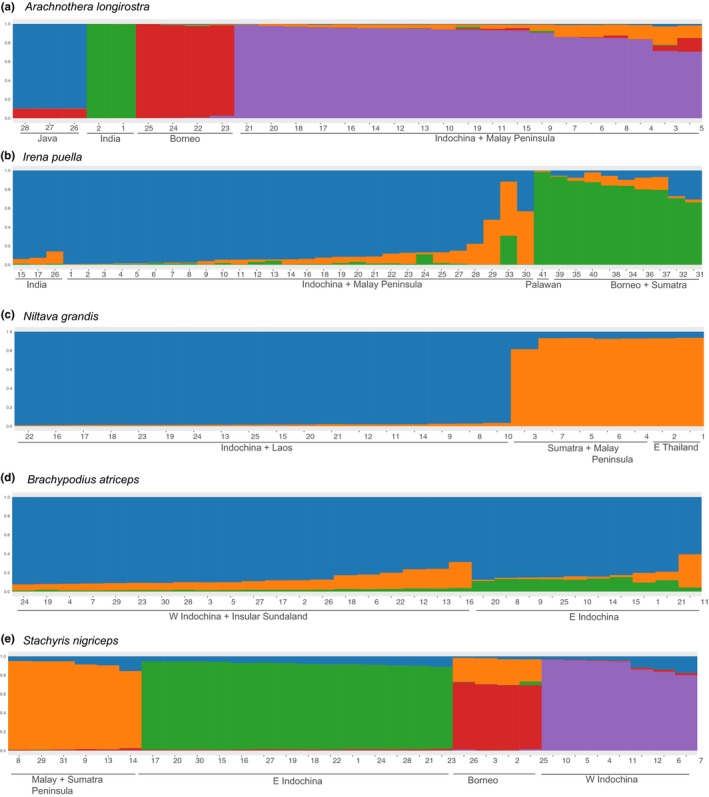
Results of Structure analysis for each of the five study species, based on the optimal *K* values (Table 2). Labels for individuals (columns) correspond to those in Table 1. Colors represent membership in different genetic clusters

In general, Structure analysis corroborates (and occasionally adds resolution to) population genetic patterns uncovered by the above analyses (Figures 3a–e and S4). For *A. longirostra*, shared genetic ancestry (red in Figure 3a) is observed between Javan and Bornean individuals, suggesting recent gene flow. In *I. puella*, Structure analysis assigns insular and mainland individuals to different groups (based on the amount of blue and green ancestries in Figure 3b), but Indian and Indochinese individuals are assigned to the same cluster, as are those from Palawan and the other Sunda Islands, reflecting the primary divergence detected in the network analysis. The one *I. puella *individual from Peninsular Malaysia (no. 33, Figure 3b) possesses a higher level of Bornean/Sumatran ancestry (green cluster), indicating an increased level of gene flow with populations on the Sunda islands. In both *A. longirostra* and *I. puella*, the orange genetic ancestry (Figure 3a,b) does not represent a geographically localized lineage. Thus, it is presumed to represent alleles still in linkage disequilibrium from a past demographic event (e.g., population expansion and population break) or following introgression from an unsampled (or currently extinct) population. In *N. grandis*, although the main north–south groups identified in previous analyses are recovered by Structure analysis, it does not distinguish individuals of southeastern Thailand from those of Sumatra and the Malay Peninsula (Figure 3c). For *B. atriceps*, Structure analysis marginally separates individuals from eastern and western Indochina based on membership in the green genetic cluster (Figure 3d). The green cluster, however, only accounts for a small proportion of the individuals' genetic makeup (average = 11.8% in eastern Indochinese individuals) and may represent minor allele frequency differences between the two groups associated with weak genetic differentiation. Finally, in *S. nigriceps*, Structure analysis produced the same four clusters as the network analysis (Figure 3e), and Bornean birds share significant ancestry with individuals from the Malay Peninsula and Sumatra.

Overall, *F*_ST_ is highest in *S. nigriceps* (0.338) and lowest in *B. atriceps* (0.061) (Table 3). Pairwise *F*_ST_ and Nei's *D* values are correlated with branch length in the phylogenetic networks. The highest pairwise *F*_ST_ values in both *A. longirostra* and *I. puella* involve the Indian populations, as opposed to the populations of Java and Palawan, respectively. In *S. nigriceps*, all pairwise *F*_ST_ and Nei's *D* values are similar (*F*_ST_: 0.279–0.434 and Nei's *D*: 0.050–0.071), indicating relatively simultaneous divergence among populations.

**Table 3:** Pairwise *F*_ST_ (upper triangle) and Nei's *D* (lower triangle) values between populations

*Arachnothera longirostra*				
Overall *F*_ST_	0.331			
	Borneo	India	Java	IC + MP
Borneo		0.484	0.287	0.152
India	0.198		0.570	0.299
Java	0.180	0.210		0.249
IC + MP	0.116	0.129	0.143	
*Irena puella*				
Overall *F*_ST_	0.217			
	Sum + Bor+Java	IC + MP	India	Palawan
Sum + Bor+Java		0.200	0.311	0.188
IC + MP	0.046		0.132	0.342
India	0.071	0.046		0.556
Palawan	0.069	0.107	0.119	
*Niltava grandis*				
Overall *F*_ST_	0.219			
	E Thailand	IC	Sum + MP	
E Thailand		0.262	0.287	
IC	0.103		0.188	
Sum + MP	0.095	0.073		
*Brachypodius atriceps*				
Overall *F*_ST_	0.061			
	W. IC + Sunda	E. IC		
W. IC + Sunda		0.061		
E. IC	0.031			
*Stachyris nigriceps*				
Overall *F*_ST_	0.338			
	Borneo	E. IC	Sum + MP	W. IC
Borneo		0.381	0.434	0.425
E. IC	0.065		0.309	0.279
Sum + MP	0.071	0.054		0.360
W. IC	0.070	0.050	0.062	

Abbreviations: BR, Borneo; E. IC, eastern Indochina; IC, Indochina; MP, Malay Peninsula; Sum, Sumatra; Sunda, Sundaland; W. IC, western Indochina.

### 3.3 Population history and demography

**Figure 4:**
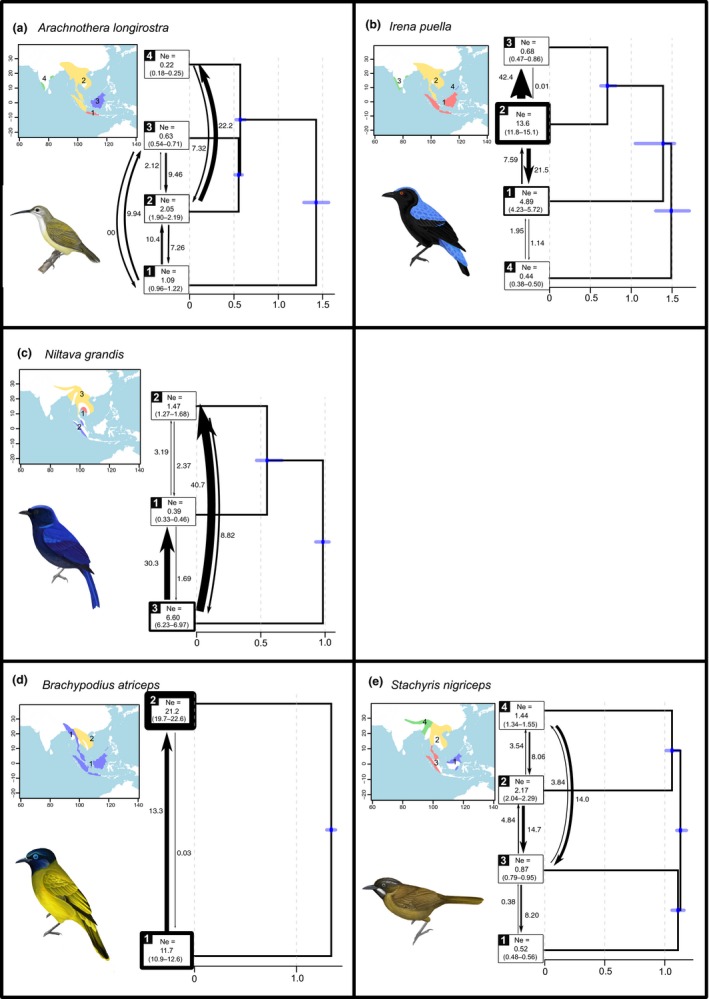
Results of G‐PhoCS analyses for each of the five study species. Each result shows the branching pattern of populations. The number of each population corresponds to the number in the inset map. The thickness of the box outline of each population corresponds to the effective population size (*N*_e_ × 10^6^) of that population. The 95% highest posterior density (HPD) interval of each *N*_e_ estimate is shown in parenthesis below each estimate. Arrows indicate gene flow rate (proportion of individuals in the receiving population that immigrated from the source population, ×10^–8^) between pairs of geographically adjacent populations. The *x*‐axes show divergence time in units of millions of years ago, and blue bar around each node indicates HPD interval of each divergence time estimate

SNAPP species trees revealed the population branching history in each species, the details of which are described below in conjunction with the demographic results (see also Figure 4). BFD* analyses most strongly support (i.e., have the least negative marginal likelihood for) the most‐split population assignment model in each species (Table 4). Within a species, the Bayes factor (2 × log_e_BF) of the best model differs by at least 40 from other models, indicating “very strong” support (Leache et al., 2014). In addition, Bayes factors indicated that incremental lumping of populations based on SNAPP tree topology resulted in increasingly poor models, thus supporting the hierarchical relationships in the SNAPP trees (Table 4).

**Table 4:** Results of BFD* analyses

	Model	Marginal likelihood (log_e_)	Bayes Factor (relative to best model)
*Arachnothera longirostra*			
1	IC + MY, BR, IN, JV	−1542.7	
2	IC + MY+BR, IN, JV	−2068.0	−1050.6
3	IC + MY+BR + IN, JV	−2968.2	−2850.9
4	IC + MY+BR + IN+JV	−3819.7	−4554.0
*Irena puella*			
1	IN, IC + MY, SM + BR+JV, PL	−1140.1	
2	IN + IC+MY, SM + BR+JV, PL	−1213.8	−147.4
3	IN + IC+MY + SM+BR + JV, PL	−1207.6	−134.9
4	IN + IC+MY + SM+BR + JV+PL	−1296.4	−312.7
*Niltava grandis*			
1	SM + MY, seTH, IC	−6766.6	
2	SM + MY+seTH, IC	−7034.4	−535.5
3	SM + MY+seTH + IC	−8758.5	−3983.7
*Brachypodius atriceps*			
1	eIC, wIC	−2446.7	
2	eIC + wIC	−2470.8	−48.2
*Stachyris nigriceps*			
1	eIC, wIC,BR, SM + MY	−5629.6	
2	eIC + wIC,BR,SM + MY	−6584.5	−1909.8
3	eIC + wIC,BR + SM+MY	−7180.0	−3100.8

Note

Models used for population delimitation are arranged from least clumped (no. 1) to most clumped. Order of population clumping follows results of SNAPP species tree analyses (Figure 4).

Abbreviations: BR, Borneo; eIC, eastern Indochina; IC, Indochina; IN, India; JV, Java; MY, Malay Peninsula; PL, Palawan; seTH, southeastern Thailand; SM, Sumatra; TH, Thailand; wIC, western Indochina.

G‐PhoCS models were based on the SNAPP tree topology and were calibrated using relative substitution rates calculated using comparison with the Moyle et al. (2016) dataset. Mantel test indicated that phylogenetic distances from that tree were highly correlated with genetic distances based on UCE data (nperm = 999, *p* = .001). Comparison of relative branch lengths across the entire tree produced an inferred relative substitution rate of 1.94 × 10^−10^ substitutions/year (*SD* = 3.14 × 10^−5^) for our UCE dataset (Figure S5). Based on inspection of MCMC trace plots with Tracer, all G‐PhoCS runs converged and displayed appropriate mixing after about 10%–35% of the total run length, which we removed as burn‐in for all parameter estimates. In general, estimates of *θ*(= 4*N*_e_ *μ*, population mutation rate) for modern (vs. inferred ancestral) populations tended to have higher ESS values compared to estimates of other parameters (e.g., migration rates, Table S1). The G‐PhoCS dataset of each species either included all the loci that passed filtering (up to 3,640) or a smaller subset (≥2,200 loci) because of computer memory issues with the program (Table 5).

**Table 5:** Number of loci, and alignment length, and population genetics metrics of UCE loci used in G‐PhoCS analyses

	*A. longirostra*				*I. puella*			
No. of UCE loci	3,047				2,500			
Avg. alignment length (bp)	297.9				244.3			
No. in Figure 4	1	2	3	4	1	2	3	4
Populations	Java	IC + MP	Borneo	India	Sum + Bor+Java	IC + MP	India	Palawan
Sample size	3	19	4	2	9	28	3	1
Avg. Watterson's theta	0.212	0.366	0.126	0.109	0.425	0.893	0.183	0.144
Avg. no. of variable sites/bp	0.001	0.005	0.001	0.001	0.006	0.016	0.002	0.001
Avg. no. exclusive variants (Sx)	0.296	1.100	0.138	0.103	0.844	3.367	0.139	0.113
Avg. no. of variants fixed in population (Sf)	0.017	0.000	0.003	0.014	0.001	< 0.001	0.002	0.066
Avg. Tajima's *D* a	—	−0.646	—	—	—	−1.493	—	—
Avg. Fu and Li's *D* a	—	−0.721	—	—	—	−2.304	—	—

	*N. grandis*			*B. atriceps*		*S. nigriceps*			
No. of UCE loci	3,640			2,200		3,523			
Avg. alignment length (bp)	300.9			258.4		290.6			
No. in Figure 4	1	2	3	1	2	1	2	3	4
Populations	SE Thailand	Sum + MP	IC	W IC	E IC	Borneo	E IC	Sum + MP	W IC
Sample size	2	5	18	20	10	4	14	6	7
Avg. Watterson's theta	0.188	0.292	0.601	1.052	0.706	0.177	0.377	0.280	0.305
Avg. no. of variable sites/bp	0.001	0.002	0.008	0.016	0.009	0.001	0.005	0.003	0.003
Avg. no. exclusive variants (Sx)	0.171	0.426	1.939	3.374	1.609	0.273	1.077	0.514	0.654
Avg. no. of variants fixed in population (Sf)	0.010	0.001	0.000	0.001	0.001	0.023	0.002	0.012	0.007
Avg. Tajima's *D* a	—	—	−1.040	−1.551	−1.099	—	−0.712	—	—
Avg. Fu and Li's *D*or D* a	—	—	−1.245	−2.251	−1.434	—	−0.831	—	—

Abbreviations: Bor, Borneo; IC, Indochina; MP, Malay Peninsula; Sum, Sumatra.

a Tajima's *D *and Fu and Li's *D* or D* statistics are calculated for populations with sample size of at least 10. Fu and Li's D* is calculated for *S. nigriceps *due to the lack of an outgroup taxon (Fu & Li, 1993; Tajima, 1989).

In the study species, the oldest divergence time between two populations is *c*. 1 myr or more (Figure 4). Peripheral or geographically restricted populations (e.g., India, Palawan) tend to have smaller *N*_e_ values, on the order of a few hundred thousand individuals. In *A. longirostra*, the Javan population diverged first (*c*. 1.4 mya), followed by almost simultaneous divergence of the remaining three populations around 0.5–0.6 mya (Figure 4a). The divergence of *I. puella *populations progressed from east to west (Figure 4b), with the Palawan population diverging first (*c*. 1.5 mya). The rate of gene flow is highest from the central Indochina/Malay Peninsula population (which also has the highest *N*_e_) into India and then the Greater Sunda Islands. In *N. grandis*, the northern Indochinese population diverged first (*c*. 1 mya), followed by the two southern populations (Figure 4c). The Indochinese population has contributed higher gene flow into the two southern populations, especially the population located in southeastern Thailand. The two *B. atriceps *populations were separated *c*. 1.4 mya, with strongly asymmetrical gene flow rate. Their estimated *N*_e_'s are about an order of magnitude larger than populations of other species (Figure 4d). Finally, in *S. nigriceps*, northern and southern lineages divided *c*. 1.1 mya, followed shortly thereafter by further splitting within the north and the south (Figure 4e). Gene flow from the two northern populations into the Malay Peninsula/Sumatra population has been 2–4 times higher than other rates of gene flow in *S. nigriceps*. Additional population genetic summary statistics are reported in Table 5.

## 4 DISCUSSION

We conducted sequence capture and high‐throughput sequencing of historical and modern DNA samples from populations of five codistributed species of Southeast Asian rainforest birds: *Arachnothera longirostra*, *Irena puella*, *Brachypodius atriceps*, *Niltava grandis*, and *Stachyris nigriceps*. Because of the large amount of data and broad sampling, the analysis provides an unprecedented comparative perspective on the range‐wide genetic structure and population history of these species. Overall, we discovered population patterns that were expected based on well‐known geographic structures (e.g., the Isthmus of Kra), some that were consistent with habitat requirements of the species (e.g., structuring in eurytopic vs. stenotopic species). Sequencing of samples from areas that are previously unstudied also yields novel insights (e.g., India populations of both *A. longirostra* and *I. puella* are nested within, but strongly differentiated from, their respective Indochinese populations). We combined coalescent analyses and large amounts of data to produce highly resolved estimates of population demography and divergence parameters. These estimates support earlier findings that are largely based on mtDNA data.

### 4.1 Impact of marker choice

Marker choice and the fit of data to evolutionary models used for analysis can impact results and inferences. The markers selected here, sequences linked to ultraconserved genomic regions, are useful because the same regions can be sequenced across taxa, reducing the systematic biases across species that are introduced by the interaction of sequence assembly algorithms with genetic diversity in datasets of nonoverlapping markers (Harvey et al., 2015). However, the conservation of UCEs is likely associated with strong purifying selection (Katzman et al., 2007), which might impact diversity at nearby variable sites used for analyses. If the impacts of purifying selection on these datasets result in a poor fit to evolutionary models used for analysis, which often assume neutrality, it may bias estimates within or across species. Accumulating evidence suggests, however, that few if any regions of the genome are free from selection (Cariou, Duret, & Charlat, 2016; Kern & Hahn, 2018). Thus, any genomic data might be subject to some degree of model misspecification. In fact, UCEs provide a better fit to some evolutionary models that assume neutrality than other markers, such as exons (Reddy et al., 2017). In addition, comparisons across species are likely reliable if all of the datasets are similarly impacted by selection. For example, both Tajima's *D* and Fu and Li's *D* are negative in all populations for which we had large samples (Table 5). Together, these results suggest that inferences within and across species from markers linked to UCEs are likely to be no more biased than those from other classes of markers or genomic regions.

### 4.2 Correspondence with hypothesized drivers of population structure in Southeast Asia

Several geographic features, events, and forces are thought to have played an important role in the diversification of Southeast Asian organisms. The effect of the most important of these on the population structure of the five target species is summarized here.

#### 4.2.1 The Isthmus of Kra as a population barrier

The north–south split that separates Indochinese and Sundaic avifaunas (and other groups of organisms) has intrigued biogeographers for generations (Wallace, 1876). The traditional view of this division is that the Isthmus of Kra (usually identified as the line connecting Chumphon and Ranong, Thailand, *c*. 10.5°N99°E), or the region just north of it, forms the interface between Indochinese and Sundaic biogeographic regions (Holt et al., 2013). This transition was thought to result from ancient marine transgressions (vicariance) at the Isthmus and/or a change in vegetation (Hughes et al., 2003; Woodruff, 2003). The former hypothesis has subsequently been rejected by Woodruff and Turner (2009); the latter is still viewed as a potentially important force in separating rainforest plant and animal species north and south (Baltzer & Davies, 2012). The vegetational transition encompasses changes from evergreen to seasonal rainforest (at the Kangar–Pattani line south of the Isthmus, *c*. 6°N, Figure 1) and from seasonal rainforest to mixed deciduous forest further north. It is driven mainly by variation in rainfall (Figure S6A; Round, Hughes, & Woodruff, 2003; van Steenis, 1950).

Using a single mtDNA gene and haplotype network reconstruction, Dejtaradol et al. (2016) examined population genetic structures in three species of lowland and montane *Pycnonotus *and *Brachypodius* bulbuls that span the Isthmus of Kra. They found, within each species, that the transition between northern and southern lineages generally occurs well into northern Thailand (>18°N), not at the Isthmus of Kra. In our study, only the two hill and montane species—*N. grandis* and *S. nigriceps*—have populations that are split at the interface between Indochina and Sundaland (Figure 2). Unfortunately, because of inadequate historical specimen coverage, our sampling in the southern Myanmar–Thai region was not dense enough to locate the precise location of the population breaks in either species (Figure S1). We can say only that the north–south split in *S. nigriceps* occurs between southern Thailand (7.0°N) and central Thailand (16.8°N). The southern *N. grandis* lineage consists of individuals from Sundaland and southeastern Thailand. Range maps indicate that this southeastern Thailand population (which also occupies southern Cambodia, including the Cardamom Mountains) is disjunct from other populations (Clement, 2019). It is also genetically distinct from other southern populations and, thus, seems to represent a relictual element of a perhaps previously more widespread southern lineage. Notably, both *N. grandis* and *S. nigriceps* occupy hill and submontane forest (c. 500–2,000 m; Robson, 2005), suggesting that the low elevation of the Isthmus may have contributed to lineage splitting or the maintenance of lineage separation in the two species.

#### 4.2.2 The east–west transition in central Indochina

Two of the target species, *S. nigriceps* and *B. atriceps*, exhibit a distinct east‐to‐west genetic break in Indochina (Figure 2). In *S. nigriceps*, the east–west divide is especially well resolved spatially. It occurs in the northern portion of the Tenasserim Range (the mountains that separates the majority of Myanmar from Thailand) or the Salween River, which runs along the western side of the Tenasserim Range into the Andaman Sea. In *B. atriceps*, the two northernmost individuals in the western Indochina group were collected in eastern Bangladesh and, therefore, the location of the split between the eastern and western groups is not as clear as in *S. nigriceps*. We suggest it may lie in the Tenasserim Range or the dry Irrawaddy Plains of central Myanmar.

The east–west genetic divide exhibited by *S. nigriceps* in Indochina was identified in the past based on species or subspecies distributions of other taxa (Deignan, 1945; Smythies, 1953). The western portion of Indochina has traditionally been classified as the Assam–Burma biogeographic subregion, and it distinguishes most of Myanmar (except for eastern Shan state) from the rest of Indochina. Recently, molecular studies have confirmed the importance of the east–west division in Indochinese birds. Reddy and Moyle (2011) found the transition corresponds to major phylogeographic breaks within both large scimitar babbler *Pomatorhinus hypoleucos* and coral‐billed scimitar babbler *P. ferruginosus*. Fuchs et al. (2015) showed that two species of *Alophoixus* bulbuls (*A. flaveolus* and *A. pallidus*) meet in secondary contact in this transition zone, after *A. pallidus* underwent putative ring speciation around the drier lowland basin of east‐central Thailand. Manawatthana et al. (2017) found that another bulbul sister pair also forms a contact zone in this region: olive bulbul *Iole viridescens*, distributed from peninsular Thailand to western Myanmar, and gray‐eyed bulbul *I. propinqua*, distributed from eastern Myanmar to Vietnam and other countries in eastern Indochina.

The historical geographic phenomena responsible for Indochina's east–west transition zone are not well established and probably multifaceted. Although the Salween River and/or the tall Tenasserim Range may form a barrier between eastern and western Indochinese taxa in the north, in the southern portion of Indochina (peninsular Myanmar and Thailand), seasonal rainfall differences as well as southern stretches of the Tenasserim Range may contribute to geographic and ecological barriers. Southwestern Indochina receives much more rainfall than the southeast during the wettest months, when the southwest monsoons are active due to the orographic effects of the Tenasserim Range (Figure S6B; Hijmans, Cameron, Parra, Jones, & Jarvis, 2005). Given the importance of the area composed of eastern Shan State (Myanmar), southern Yunnan (China), and northwestern Thailand as an area of potential secondary contact between eastern and western lineages, it needs to be studied in greater detail from the perspective of avian hybridization.

#### 4.2.3 Island connection and disconnection in Sundaland

The Greater Sunda Islands and the mainland experienced repeated connection and disconnection during the Pleistocene because of eustatic sea‐level changes associated with periodic global glaciation events (Whitmore, 1981). Corresponding to these climatic events, habitat type, position, and coverage changed dynamically both on and between the islands (Cannon, Morley, & Bush, 2009; Morley, 2012). These dynamics are known to have influenced rainforest bird populations in variable ways, resulting in (a) little structure among some Sundaic populations because of gene flow during land‐bridge connections, (b) substantial structure among some island populations because of presumed habitat barriers (forests with relatively open canopy) across land bridges or dispersal limitation, (c) substantial population structure within Borneo because of early Pleistocene isolation followed by presumed more recent dispersal and secondary contact, and (d) substantial variation among and within islands because of paraphyly or pre‐Pleistocene divergence among populations/species (Lim et al., 2017, 2011; Moyle et al., 2011; Sheldon et al., 2009).

Our current study finds some concordance between population structure in Sundaland, and dispersal ability and habitat requirements of species. Across Sundaland, *B. atriceps* exhibits the least amount of structure. It is a partially frugivorous species that often forages along forest edges and may thus be more vagile than the other study species (Fishpool & Tobias, 2005). The lack of genetic distinctiveness is true even for the sole individual representing the *baweanus *species, which is a gray‐morph bird (USNM 181552; no. 12, Figure S2) collected from Bawean Island in the Java Sea. The Bawean Island lies on the Sunda Shelf and was connected to other Sunda landmasses when sea level was approximately 50 m below current level (Voris, 2000). The other two eurytopic species (*A. longirostra* and *I. puella*) show more pronounced population structure in Sundaland, with peripheral Sunda islands (Palawan and/or Java) containing populations that diverged the earliest (Figure 4). Although we did not include Palawan *A. longirostra *in our analysis because of poor sequencing output, previous studies have shown that Palawan contains the most divergent *A. longirostra *population (Moyle et al., 2011; Rahman, Gawin, & Moritz, 2010). Thus, the intermediate vagility and habitat breadths of *A. longirostra* and *I. puella* (compared to *B. atriceps* and the two hill/submontane species) might have encouraged divergence of island populations through a combination of an ability to colonize islands while remaining relatively isolated (see gene flow rates in Figure 4) after colonization (Claramunt, Derryberry, Remsen, & Brumfield, 2012).

### 4.3 Implications of divergence times and demographic histories

The timing of the deepest splits within our study species ranged from *c*. 1 to 1.5 mya (Figure 4). Such large within‐species divergence values are not unusual in the tropics (Smith, Seeholzer, Harvey, Cuervo, & Brumfield, 2017), including Southeast Asia. For example, within the bulbul species *Iole propinqua* (eastern Indochina) and *I. viridescens* (western Indochina), the deepest estimated population divergence times are 0.9 and 1.7 mya, respectively (Manawatthana et al., 2017). Similarly, between subspecies of *Alophoixus ochraceus* in the Thai–Malay peninsula and eastern Thailand/Vietnam, the divergence time is estimated to be 1.2 mya (Fuchs et al., 2015). Leonard et al. (2015) compared 28 phylogeographic studies focused on rainforest mammal and bird taxa that are distributed primarily in Sundaland and found interpopulation divergence times spanned the Pleistocene, with the oldest bird splits ranging from *c*. 1.4 to 2.6 mya between populations on Borneo versus the Malay Peninsula/Sumatra. For the short‐tailed babbler *Trichastoma malaccense*, Lim and Sheldon (2011) estimated the time of divergence between the northeastern Borneo population and the rest of the species to be *c*. 3.8 mya. Of course, morphological crypsis and taxonomic subjectivity in splitting species probably come into play in such extreme cases.

The timing and topology of population divergence based on our UCE data are generally corroborated by previous molecular genetics studies of the same species, when such data exist. Using 10 nuclear loci and two mtDNA genes, Lim and Sheldon (2011) estimated the divergence time between *A. longirostra* populations of Borneo and the Malay Peninsula to be *c*. 0.6 mya. Here, using a broader sampling of individuals in mainland Southeast Asia, we found the divergence time to be about the same (Figure 4a). Estimates of gene flow rate and effective population sizes are also of similar magnitude in the two studies. Interestingly, an almost simultaneous divergence of *c*. 0.6 mya separates *A. longirostra* populations into Indian, Indochinese, and Bornean lineages. The split of the India population of *I. puella *from that in Indochina dates to a similar time period of *c*. 0.7 mya. This temporal coincidence suggests a large‐scale event (probably climatic) drove the breakups in both species.

Using mitochondrial data, Moltesen, Irestedt, Fjeldsa, Ericson, and Jonsson (2012) estimated that Palawan's *I. puella* population (subspecies *tweeddalei*) diverged from other populations *c*. 2 mya (vs. *c*. 1.5 mya obtained in our study). Although Palawan is part of the Sunda Shelf, its connection with the rest of the Sunda islands is tenuous due to the deep sea channel (140 m) that permanently separates it from Borneo, even during periods of extremely low sea level (Esselstyn et al., 2010; Lim et al., 2014). Palawan came to its current location northeast of Borneo *c*. 10 mya (Hall, 2009) but likely experienced periodic rainforest contraction, even as recently as 21 kya (Wurster et al., 2010), which would have affected population demography of its rainforest species. Lim et al. (2014) reviewed mtDNA divergence levels between pairs of avian sister taxa found on northern Borneo and Palawan, and they fell mainly into two groups: deep (7.9%–14%) and shallow (0.3%–0.9%). The divergence level of *I. puella* appears to be intermediate, similar to the rufous‐tailed tailorbird *Orthotomus sericeus* (mtDNA divergence 1.7%; divergence time estimated from eight loci at 1.2 mya, as opposed to 1.5 mya in *I. puella*). Therefore, despite the long‐term separation of Palawan from the rest of Sundaland, some dispersive species appear to have moved between it and Borneo in the intervening years.

The two main lineages of *N. grandis* (north vs. south) diverged from one another *c*. 1 mya. There are no comparable data for this species, but judging from phylogeographic studies of other SE Asian avian taxa with similar distributions, this level of divergence is typical. In the bulbul species complex comprising *Alophoixus ochraceus/pallidus*, for example, the populations on the Malay Peninsula/Borneo are separated from the populations in Indochina by *c*. 1.1 mya (Fuchs et al., 2015). A corresponding split in the *Pycnonotus melanicterus* bulbul species group, however, is dated at only *c*. 0.4 mya (Dejtaradol et al., 2016).

The east–west divide of *B. atriceps* is deep (1.4 mya), despite a low relative measure of population differentiation (*F*_ST_ = 0.061). This disparity may be caused by this species' unusually large *N*_e_, which would have slowed the rate of genetic drift. Using mtDNA data, Dejtaradol et al. (2016, Figure 3d) estimated the same split to be around 3 mya, but this assumes their northern lineage is equivalent to our eastern Indochina lineage. Within Sundaland, the genetic similarity of *B. atriceps* populations was first noted by Chua et al. (2015) using mtDNA ND2 sequences. The only exception was the population (subspecies = *hodiernus*, Figure 1) on Maratua Island (an oceanic island off the east coast of Borneo separated by a permanent sea barrier 200 m deep), which is >2% divergent from other Sundaic populations. The almost concurrent divergence of the four *S. nigriceps* lineages suggests the presence of expansive suitable habitats *c*. 1.3 mya ago, followed by rapid vicariance events. As in *A. longirostra*, these closely timed events may be related to large‐scale climatic changes. Although our estimates of divergence times largely agree with previous available studies, the comparability of absolute‐timing estimates from different studies depends on factors such as sample size, actual samples and genetic markers used, and assumptions related to substitution rates of the markers and generation times of the study species (e.g., Winker, Glenn, & Faircloth, 2018).

### 4.4 Future prospects for Southeast Asian phylogeography

Because of poor sampling in Southeast Asia, species‐level studies usually present incomplete pictures of population structure and history across the region. To ameliorate this problem, researchers have turned to comparing mtDNA from historical museum specimens, but mtDNA comparisons can yield incorrect measures of genealogical relationships and they generally provide limited population genetic information (Funk & Omland, 2003). By applying next‐generation sequencing techniques to historical DNA extracted from museum specimens, as well as modern samples, our study represents a breakthrough for range‐wide phylogeographic studies in Southeast Asia. We were able to substantially improve geographic sampling and sampling of the genome. However, we still have a long way to go to achieve an in‐depth understanding of Southeast Asian phylogeographic history. Facilities and expertise for conducting high‐throughput DNA sequencing on historical samples are currently limited, and the cost is likely prohibitive for many researchers (Bi et al., 2013). Moreover, although we managed to use historical specimens in our study, the quality of DNA from these samples is naturally inferior to that from freshly collected samples. Historical museum specimens produce sequences that are often fragmentary and require substantial error correction. Further, although existing museum specimens improve geographic coverage, they may still represent patchy or biased samples of species' distributions. We believe strongly that extensive modern sampling is required, sampling that preserves as much information from the specimens as possible (e.g., skeletal and muscular structure, stomach contents, parasites, microbiomes, RNA molecules, proteins, soft parts) and which may be applied to studies of diet, parasitology, toxicology, epidemiology, etc., as well as helping to solve phylogeographic issues (Webster, 2017). Modern collections are essential not only for the study of evolution and systematics, they also become important snapshots in time as Southeast Asia experiences unprecedented changes to its natural environment.

## CONFLICT OF INTEREST

None declared.

## AUTHOR CONTRIBUTIONS

H.C.L. conceived and designed the project and conducted molecular laboratory work; H.C.L., S.B.S., and M.G.H. analyzed the data; H.C.L., F.H.S., S.B.S., and M.G.H wrote the manuscript with input from all other authors; F.H.S and R.G.M. provided modern tissue samples; and M.J.B. and R.C.F. contributed laboratory protocols and reagents.

## Data Availability

Pseudoreference genome fasta files and BAM files are available from Dryad Digital Repository: https://datadryad.org/resource/doi:10.5061/dryad.2c220. Custom scripts are available from https://github.com/hawchuan/PythonScripts.
