# Supplementary material for: Opening the door to greater phylogeographic inference in Southeast Asia: Comparative genomic study of five codistributed rainforest bird species using target capture and historical DNA
Source: Ecol Evol. 2020 Mar 6;10(7):3222–47. doi: 10.1002/ece3.5964 (PMC7141000; doi:10.1002/ece3.5964)
Supplement: Supplementary file 1 [file ECE3-10-3222-s001.pdf]

Supporting Information

Opening the door to greater phylogeographic inference in Southeast Asia: Comparative study of five co-distributed rainforest bird species using target capture and historical DNA

HAW CHUAN LIM, SUBIR B. SHAKYA, MICHAEL G. HARVEY, ROBERT G. MOYLE, ROBERT C. FLEISCHER, MICHAEL J. BRAUN, AND FREDERICK H. SHELDON

Table S1 Effective sample sizes of parameters estimated by G-PhoCS. Bor = Borneo, IC = Indochina, MP = Malay Peninsula, Sum = Sumatra.

Fig. S1 Map of southern and southeastern Asia showing locations (red triangles) of all target species samples used in this study. The sampled subregions are: 1 - India, 2 - Himalayan foothills, 3 - Chin Rakhine, 4 - Kachin Sagaing, 5 - C Myanmar, 6 - Shan, 7 - E Myanmar, 8 - N Thailand, 9 - N C Laos, 10 – Tonkin-Guangxi, 11 - Annam, 12 - S Laos, 13 - Cambodia, 14 - Cochinchina, 15 - NE Thailand, 16 - E Thailand, 17 - C Thailand, 18 - Tanintharyi, 19 - Malay Peninsula, 21 - E Sumatra, 22 - W Sumatra, 23 - Brunei & Sarawak, 24 - Sabah, 25 - Palawan, 26 - E Kalimantan, 27 - W C S Kalimantan, and 28 – Java.

Fig. S2 Phylogenetic network and PCA plot for each of the study species (A-E). In each phylogenetic network, the color of each cluster of individuals corresponds to the color in the inset map. Numbers of individuals and names of clusters correspond to those in Table 1. IC = Indochina, MP = Malay Peninsula, Sum = Sumatra, Sunda = Sundaland.

Fig. S3 Results of Discriminant Analysis of Principal Components (DAPC). PC = principal component, DF = discriminant function, IC = Indochina, MP = Malay Peninsula, Sum = Sumatra, Thai = Thailand.

Fig. S4 Results of additional Structure analyses using additional *K* values ($\pm$ 1 of the optimal *K* for each species).

Fig. S5 (A) Scatterplot of the number of substitutions and divergence times. Each circle presents one pairwise comparison between two oscine taxa in the time-calibrated phylogenetic tree of Moyle *et al.* (2016), (B) Histogram of substitution rates based on number of substitutions divided by divergence time.

Fig. S6 Amount of rainfall (mm) in the driest (A) and wettest (B) months based on the WorldClim v 1.4 dataset (Hijmans, Cameron, Parra, Jones, & Jarvis, 2005).

REFERENCES

Hijmans, R. J., Cameron, S. E., Parra, J. L., Jones, P. G., & Jarvis, A. (2005). Very high resolution interpolated climate surfaces for global land areas. *International Journal of Climatology*, *25*, 1965-1978.

Moyle, R. G., Oliveros, C. H., Andersen, M. J., Hosner, P. A., Benz, B. W., Manthey, J. D., . . . Faircloth, B. C. (2016). Tectonic collision and uplift of Wallacea triggered the global songbird radiation. *Nature Communications*, *7*, 12709.

Supplemtal Table 1

Effective sample size of each of each G-PhoCS parameter

Arachnothera longirostra

No. of MCMC runs	6

Population	Letter
India	A
IC + MP	B
Borneo	C
Java	D

Parameter	ESS
theta_A	444
theta_B	413
theta_C	595
theta_D	953
theta_BC	146
theta_ABC	261
theta_root	258
tau_BC	236
tau_ABC	187
tau_root	225
m_A->B	370
m_B->A	266
m_B->C	275
m_C->B	378
m_B->D	193
m_D->B	576
m_C->D	203
m_D->C	352
Data-ld-ln	219
Full-ld-ln	1001

Irena puella

No. of MCMC runs	5

Population	Letter
Sum+Bor+Java	A
IC+MP	B
India	C
Palawan	D

Parameter	ESS
theta_A	136
theta_B	126
theta_C	113
theta_D	456
theta_BC	91
theta_ABC	89
theta_root	125
tau_BC	93
tau_ABC	90
tau_root	92
m_A->B	108
m_B->A	93
m_B->C	96
m_C->B	161
m_A->D	162
m_D->A	167
Data-ld-ln	109
Full-ld-ln	275

Niltava grandis

No. of MCMC runs	6

Population	Letter
SE Thailand	A
Sum+MP	B
IC	C

Parameter	ESS
theta_A	217
theta_B	332
theta_C	344
theta_AB	124
theta_root	164
tau_AB	109
tau_root	153
m_A->B	143
m_B->A	153
m_A->C	135
m_C->A	204
m_B->C	135
m_C->B	209
Data-ld-ln	481
Full-ld-ln	623

Brachypodius atriceps

No. of MCMC runs	4

Population	Letter
E IC	A
W IC	B

Parameter	ESS
theta_A	1596
theta_B	1368
theta_root	774
tau_root	501
m_A->B	271
m_B->A	123
Data-ld-ln	596
Full-ld-ln	1973

Stachyris nigriceps

No. of MCMC runs	5

Population	Letter
Bor	A
E IC	B
Sum+MP	C
W IC	D

Parameter	ESS
theta_A	933
theta_B	485
theta_C	298
theta_D	404
theta_AC	141
theta_BD	114
theta_root	562
tau_AC	172
tau_BD	152
tau_root	310
m_A->C	215
m_C->A	386
m_B->C	180
m_C->B	163
m_B->D	131
m_D->B	172
m_C->D	154
m_D->C	189
Data-ld-ln	135
Full-ld-ln	702

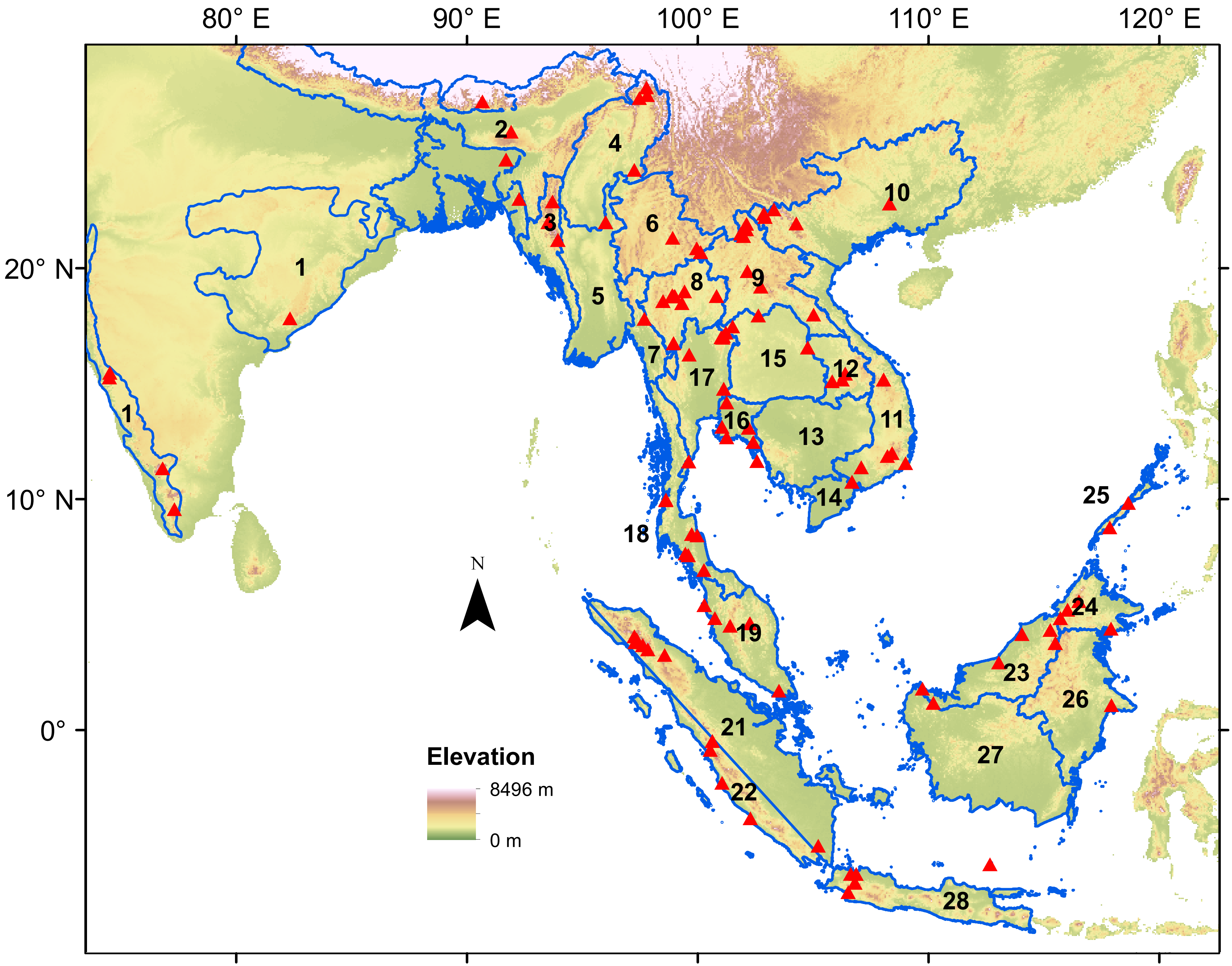

80° E
90° E
100° E
110° E
120° E
20° N
10° N
0°
N
Elevation
8496 m
0 m
1
2
3
4
5
6
7
8
9
10
11
12
13
14
15
16
17
18
19
21
22
23
24
25
26
27
28

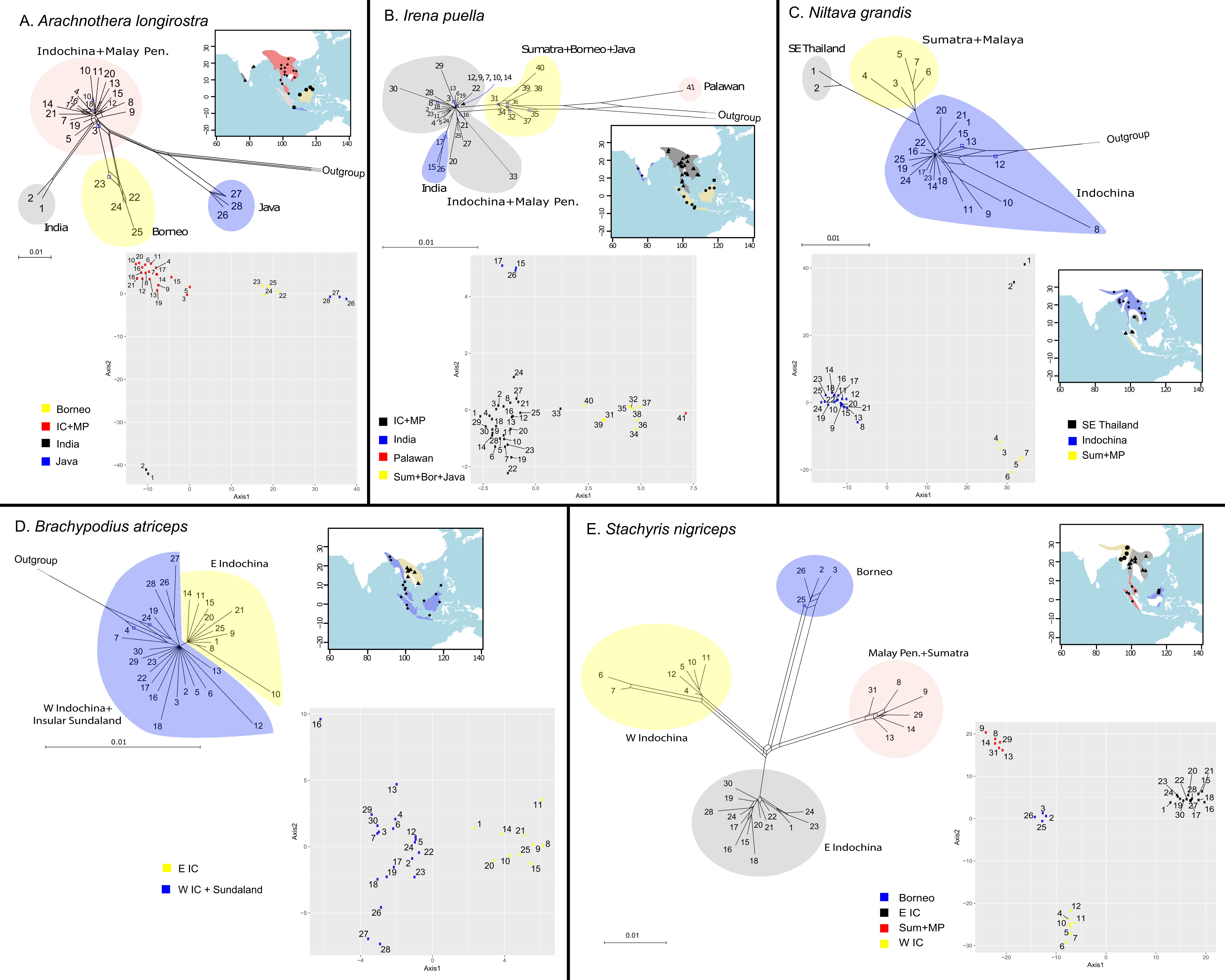
A. Arachnothera longirostra
Indochina+Malay Pen.
India
Borneo
Java
Outgroup
0.01
Borneo
IC+MP
India
Java
Axis1
Axis2
B. Irena puella
Sumatra+Borneo+Java
Palawan
Outgroup
India
Indochina+Malay Pen.
0.01
IC+MP
India
Palawan
Sum+Bor+Java
Axis1
Axis2
C. Niltava grandis
SE Thailand
Sumatra+Malaya
Outgroup
Indochina
0.01
SE Thailand
Indochina
Sum+MP
Axis1
Axis2
D. Brachypodius atriceps
Outgroup
E Indochina
W Indochina+
Insular Sundaland
0.01
E IC
W IC + Sundaland
Axis1
Axis2
E. Stachyris nigriceps
Borneo
Malay Pen.+Sumatra
W Indochina
E Indochina
0.01
Borneo
E IC
Sum+MP
W IC
Axis1
Axis2

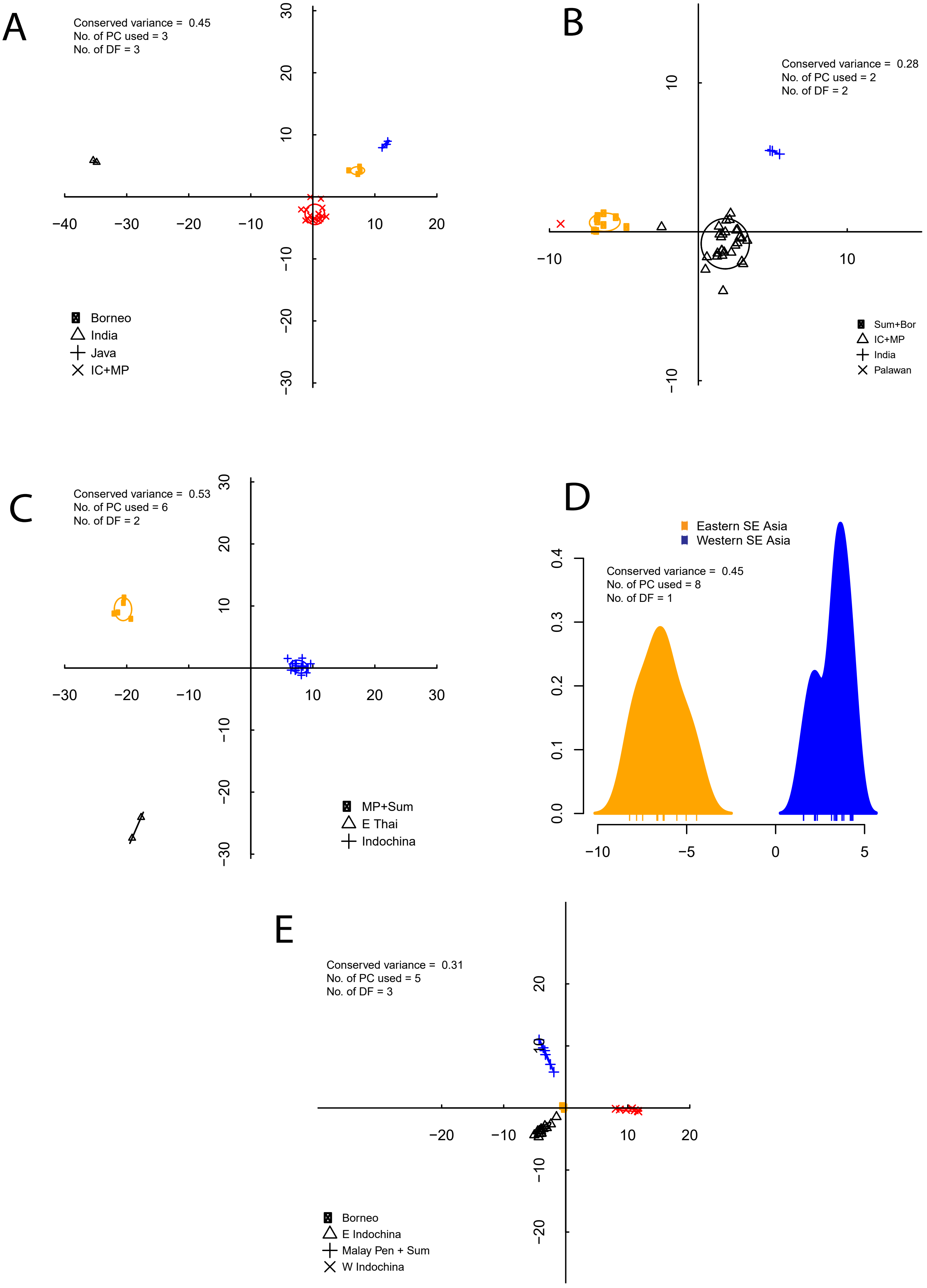
A
Conserved variance = 0.45
No. of PC used = 3
No. of DF = 3
Borneo
India
Java
IC+MP
B
Conserved variance = 0.28
No. of PC used = 2
No. of DF = 2
Sum+Bor
IC+MP
India
Palawan
C
Conserved variance = 0.53
No. of PC used = 6
No. of DF = 2
MP+Sum
E Thai
Indochina
D
Eastern SE Asia
Western SE Asia
Conserved variance = 0.45
No. of PC used = 8
No. of DF = 1
E
Conserved variance = 0.31
No. of PC used = 5
No. of DF = 3
Borneo
E Indochina
Malay Pen + Sum
W Indochina

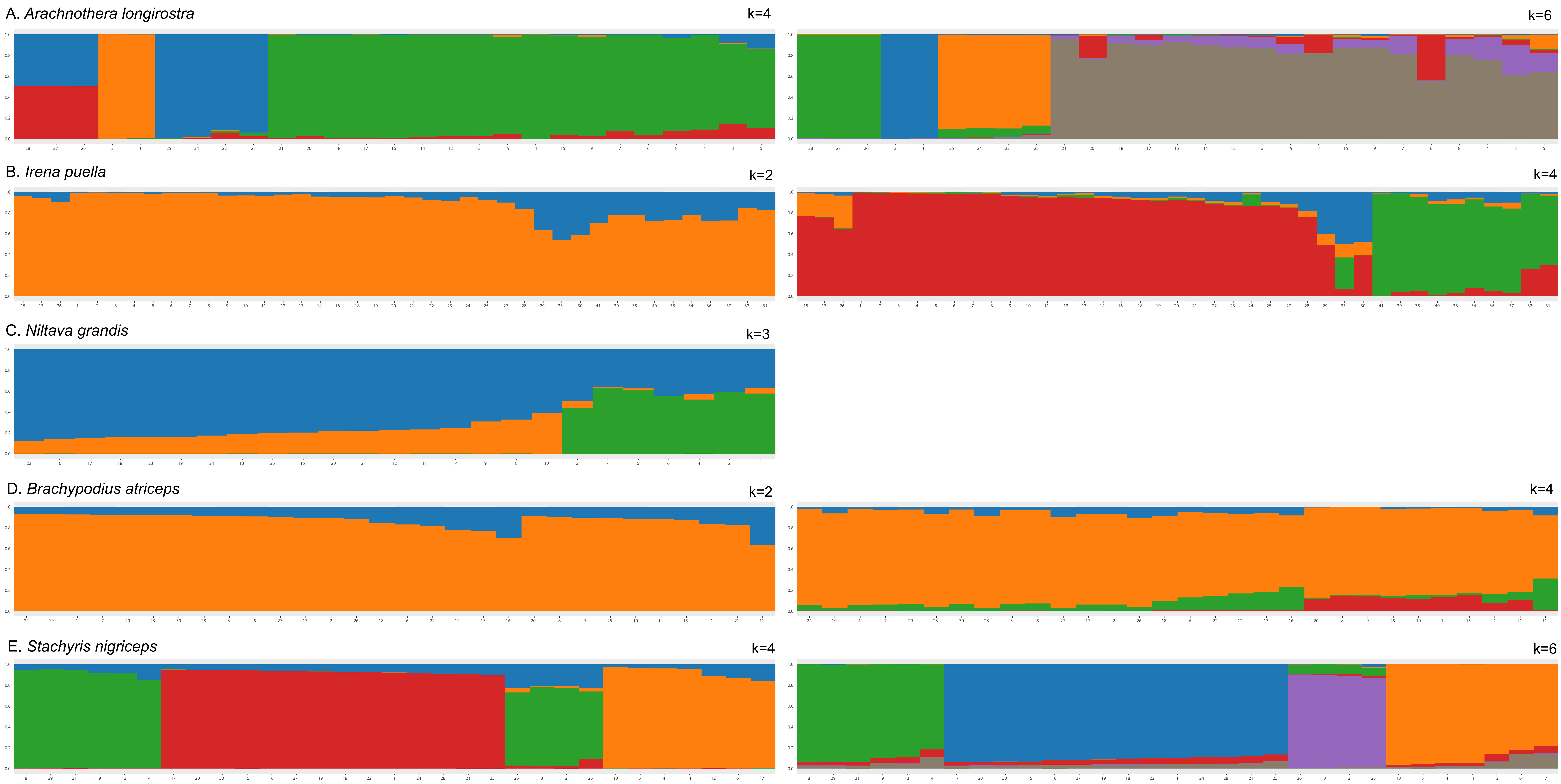
A. *Arachnothera longirostra*
k=4
k=6
B. *Irena puella*
k=2
k=4
C. *Niltava grandis*
k=3
D. *Brachypodius atriceps*
k=2
k=4
E. *Stachyris nigriceps*
k=4
k=6

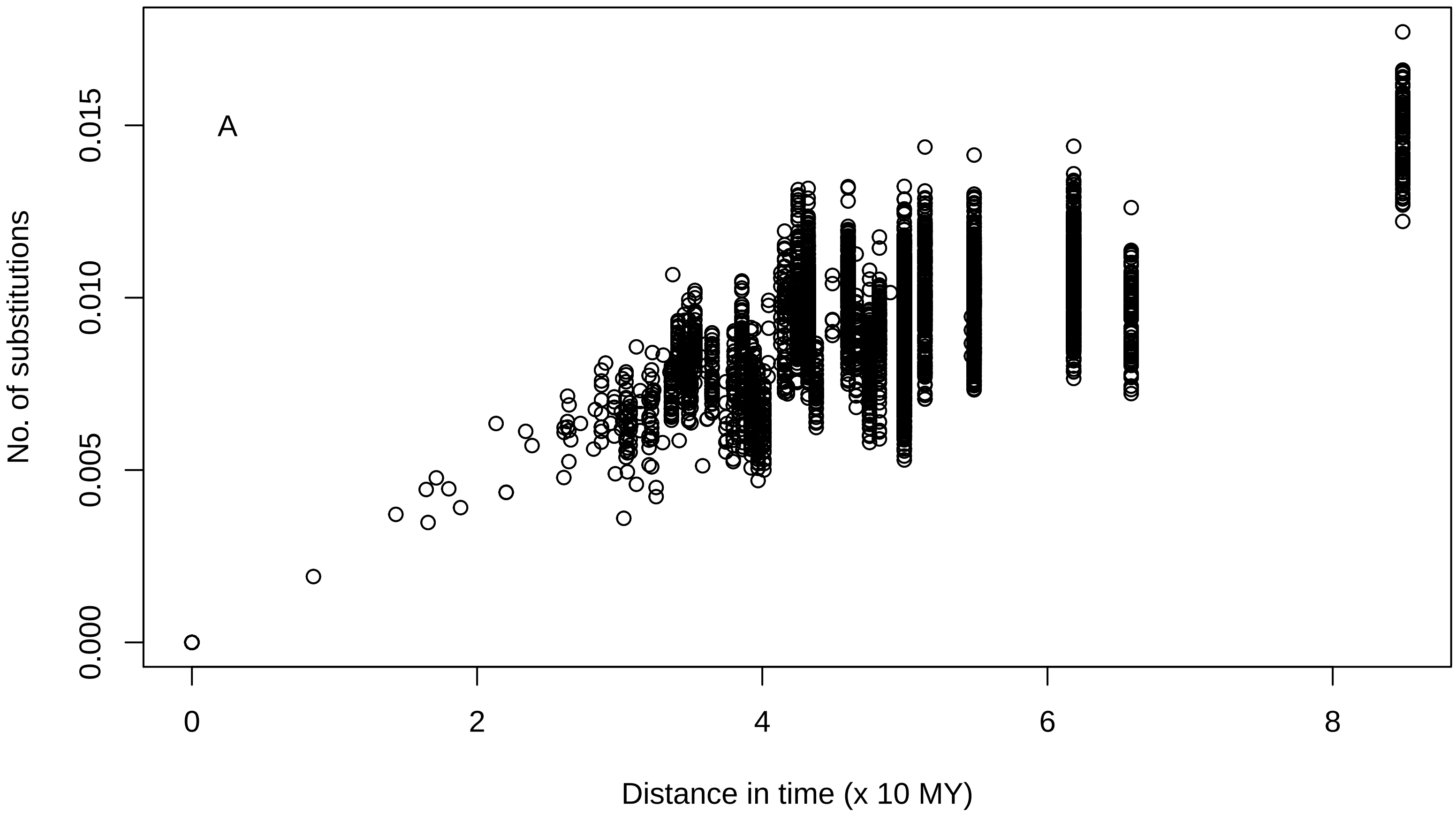

A
No. of substitutions
0.000
0.005
0.010
0.015
0
2
4
6
8
Distance in time (x 10 MY)

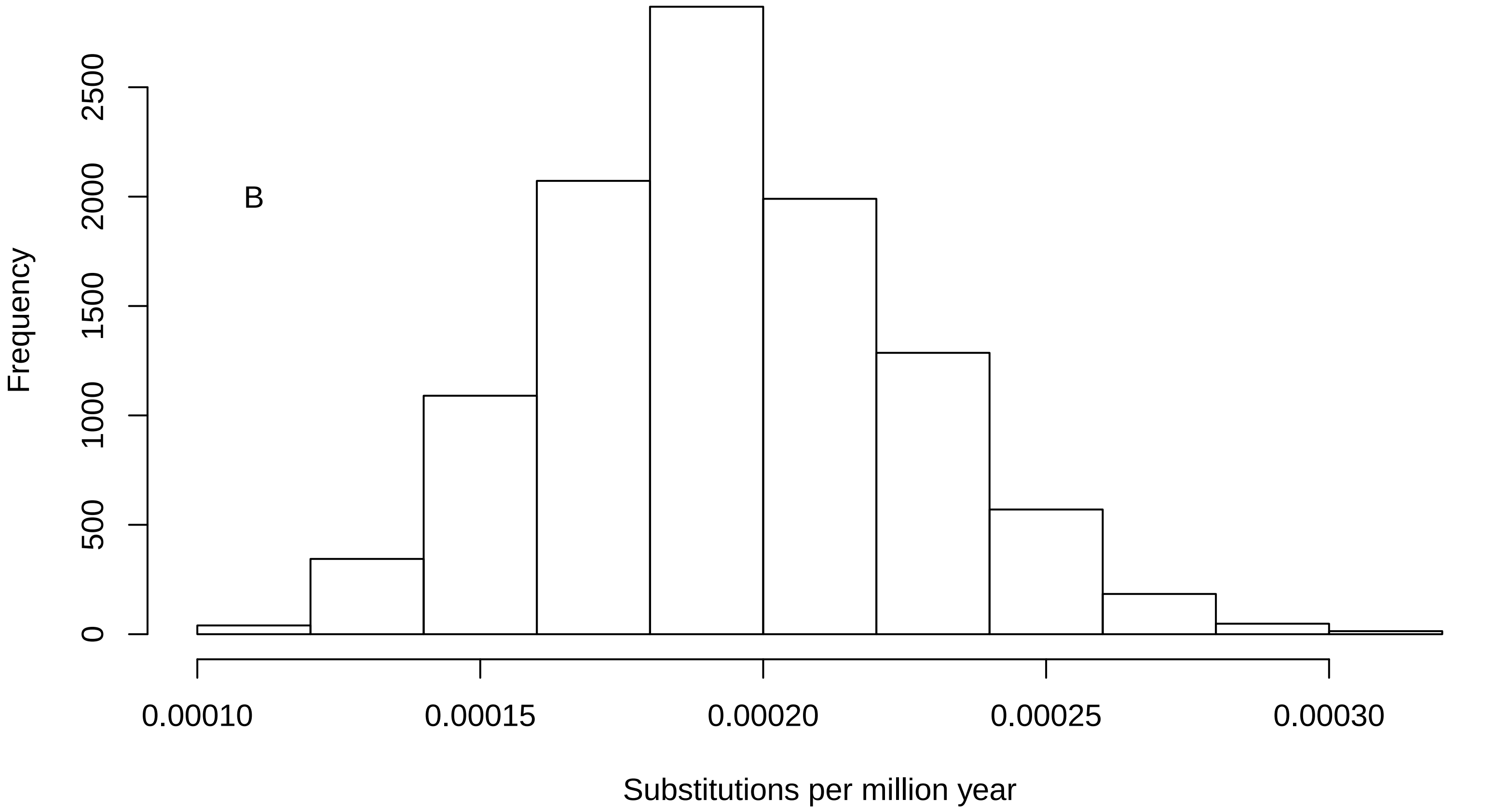

B
Frequency
0
500
1000
1500
2000
2500
0.00010
0.00015
0.00020
0.00025
0.00030
Substitutions per million year

A.

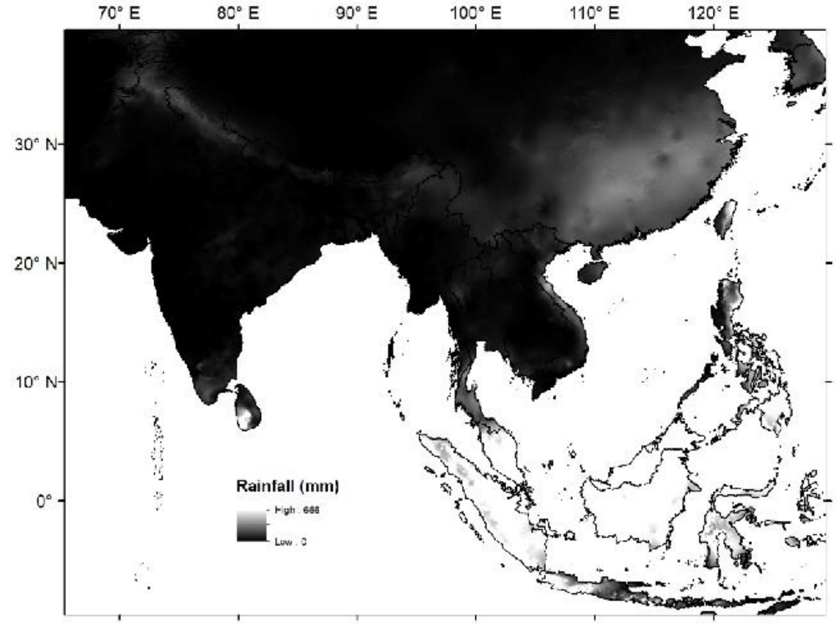
70° E
80° E
90° E
100° E
110° E
120° E
30° N
20° N
10° N
0°
Rainfall (mm)
High : 666
Low : 0

B.

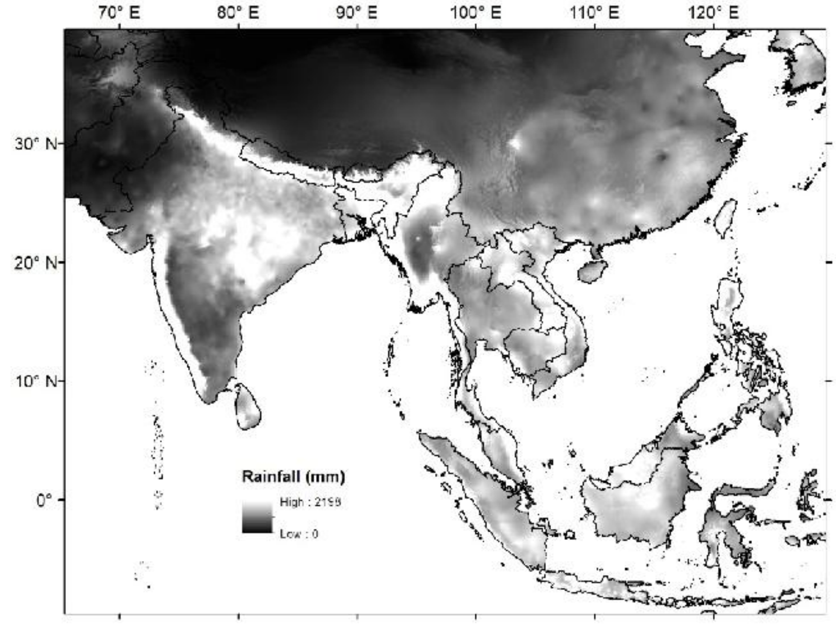
70° E
80° E
90° E
100° E
110° E
120° E
30° N
20° N
10° N
0°
Rainfall (mm)
High : 2198
Low : 0
